# Molecular and Structural Basis of Cross-Reactivity in *M. tuberculosis* Toxin–Antitoxin Systems

**DOI:** 10.3390/toxins12080481

**Published:** 2020-07-29

**Authors:** Himani Tandon, Akhila Melarkode Vattekatte, Narayanaswamy Srinivasan, Sankaran Sandhya

**Affiliations:** 1 Molecular Biophysics Unit, Indian Institute of Science, Bangalore 560012, India; himani@iisc.ac.in (H.T.); akhila17589@gmail.com (A.M.V.); 2 Biologie Intégrée du Globule Rouge UMR_S1134, INSERM, Université Paris, Université de la Réunion, Université des Antilles, F-75739 Paris, France; 3 Laboratoire d’Excellence GR-Ex, F-75739 Paris, France; 4 Faculté des Sciences et Technologies, Saint Denis Messag, F-97715 La Réunion, France; 5 Institut National de la Transfusion Sanguine (INTS), F-75739 Paris, France

**Keywords:** toxin–antitoxin, *M. tuberculosis*, bacteria, pathogenesis, protein–protein interactions, cross-talk, protein interface

## Abstract

*Mycobacterium tuberculosis* genome encodes over 80 toxin–antitoxin (TA) systems. While each toxin interacts with its cognate antitoxin, the abundance of TA systems presents an opportunity for potential non-cognate interactions. TA systems mediate manifold interactions to manage pathogenicity and stress response network of the cell and non-cognate interactions may play vital roles as well. To address if non-cognate and heterologous interactions are feasible and to understand the structural basis of their interactions, we have performed comprehensive computational analyses on the available 3D structures and generated structural models of paralogous *M. tuberculosis* VapBC and MazEF TA systems. For a majority of the TA systems, we show that non-cognate toxin–antitoxin interactions are structurally incompatible except for complexes like VapBC15 and VapBC11, which show similar interfaces and potential for cross-reactivity. For TA systems which have been experimentally shown earlier to disfavor non-cognate interactions, we demonstrate that they are structurally and stereo-chemically incompatible. For selected TA systems, our detailed structural analysis identifies specificity conferring residues. Thus, our work improves the current understanding of TA interfaces and generates a hypothesis based on congenial binding site, geometric complementarity, and chemical nature of interfaces. Overall, our work offers a structure-based explanation for non-cognate toxin-antitoxin interactions in *M. tuberculosis*.

## 1. Introduction

For a long time, the general function of toxin–antitoxin (TA) systems, was believed to be only that of plasmid addiction. However, in the past decade, other roles have been proposed for these systems [1,2,3,4]. While many studies have reported their role in persistence and cell death [5,6,7], it is not clear to what extent these interactions are coupled in the context of their cellular function. Presence of more than 80 TA systems in *Mycobacterium tuberculosis* raises the possibility of a complex network of interactions among TA pairs and their cellular targets [8]. Additionally, an intriguing and exciting possibility is cross-talk (or cross- interactions) among non-cognate toxins and antitoxins. By cross-talk, we mean that if toxins X_1_ and X_2_ and their respective cognate antitoxins Y_1_ and Y_2_ are expressed in the cell at the same time and share similar features, then in principle, Y_1_ can neutralize X_2_ or Y_2_ can neutralize X_1_. While the cognate interactions between X1 and X2 and Y1 and Y2 are expected, non-cognate or cross interaction between X1–Y2 or X2–Y1 are generally speculative. It is not fully understood whether the phenomenon of cross-talk is beneficial to the bacteria. Physical evidence of such interactions is sparse and include reports of chimeric MazF toxins in *Escherichia coli* that could activate endogenous MazF_K-12._ This is mediated through binding of the chimera to MazE_K-12_ antitoxin, likely due to a competition with the endogenous MazF_K-12_ toxin [9]. Such non-cognate interactions could disturb the delicate balance between cognate TA pairs and could result in accumulation of free toxin in the cell. It has been speculated that such non-native interactions may even lead to degeneration of these systems [8]. Nevertheless, although the possibility and existence of cross-talk seem feasible, very little experimental evidences are currently available to demonstrate their existence.

Many studies in the past have attempted to capture cross-talk between toxin–antitoxin systems in various organisms and it appears that antitoxins are very specific to their cognate toxins. For example, no cross-talk was observed between toxins and antitoxins of VapBC systems in *M. tuberculosis* [10]. However, when mutations were introduced in the wild-type VapB1 and VapB2 antitoxins from non-typeable *Haemophilus influenzae*, the mutant antitoxins showed relaxed specificity and could neutralize the non-cognate toxins [11]. Direct observation of cross-talk has so far been reported among RelBEs, MazEFs, and VapBC-MazEF systems in *M. tuberculosis.* It was observed that RelBs neutralize non-cognate RelEs through direct interactions [12]. Cross-talk has been reported between MazE9-F6 from *M. tuberculosis* [13]. Interestingly, in their study, Zhu et al. observed cross-interactions between VapB40–MazF6, VapB27–MazF6, VapB40–MazF9, and VapB27–MazF9; thereby, suggesting that toxins and antitoxins from different families can physically interact [13]. However, Ramirez et al. did not observe interactions among any of the MazEFs and suggested that cross-talk via interactions is unlikely to occur for this family of Tas, especially MazF6 [14]. Although more recently, cross-talk was reported between MazF7 and MazE9 from *M. tuberculosis* and for four SprG/SprF type I toxin-antitoxin in *Staphylococcus aureus* [15,16]. Additionally, physical interplay between cognate and non-cognate MazEF and RelBE systems from *Bifidobacterium longum* has also been reported [17]. While the Kis antitoxin from *E. coli* has been shown to inhibit CcdB, albeit with lower efficiency than cognate CcdA, the reverse was not observed [18]. Taken together, despite the presence of multiple TA systems in different organisms, cross-talk has been reported for very few of them, including *M. tuberculosis.* More experiments need to be conducted to convincingly demonstrate that this is a commonly occurring phenomenon. The growing interest in understanding the molecular basis of toxin–antitoxin interactions has seen a steady rise in the number of toxin–antitoxin structures in the protein data bank. As many as 85 non-redundant structures of cognate TA complexes from various organisms are available in the protein data bank (PDB) [19]. The availability of such structural templates provides an opportunity to analyze interactions between homologous toxin and antitoxin pairs of unknown structure. In this study, we have performed computational analyses on available 3D structures and generated homology models of cognate *M. tuberculosis* TA systems to understand the structural basis of their interactions and employed these rules to generate hypothesis on their potential for cross-talk. Through detailed analysis of structural regions of physical binding (interface) between a toxin and an antitoxin we studied the underlying rules that define their interaction. Next, we probed for the potential for cross-talk/cross-reactivity by computing the energetics involved with the structural models of non-cognate TA complexes. In addition, we have performed detailed evaluation of the stereochemical compatibility at the interface of non-cognate TA complexes. We predict that the TA interfaces of VapBC11–VapBC15 and VapBC4–VapBC5 systems are conducive to non-cognate interactions. On the other hand, a majority of the non-cognate TA systems appear unamenable to form a complex due to incompatibility at the TA interfaces. Our detailed studies are useful to capture specificity conferring residues for select TA systems studied here. We anticipate that our findings will be useful in the design of mutations that could relax the specificity of cognate TA interactions in *M. tuberculosis.* We believe that our methodology is simple and can be extended to TA systems from other organisms as and when more structures that are either experimentally solved or confidently modeled become available. We also discuss the challenges involved in modelling TA systems for such studies.

## 2. Results

### 2.1. Comparisons of TA Interfaces Predict Cases for Potential Cross-Talk and Reveal Reasons for Insulation in Others 

**Figure 1:**
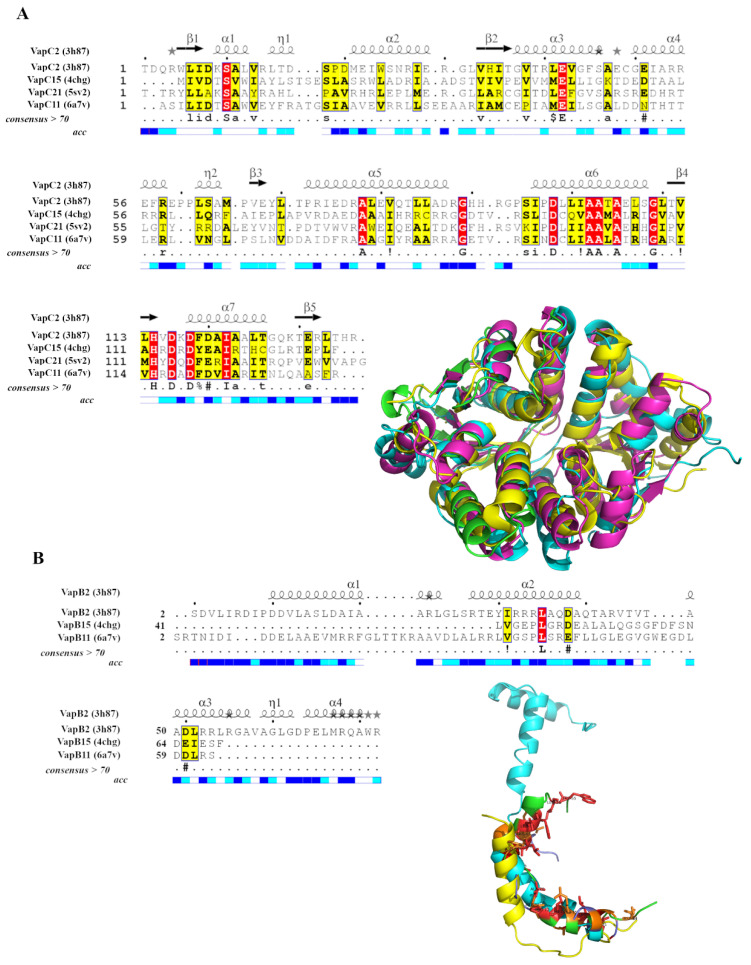
Alignments of VapC toxins and VapB antitoxins. (**A**) Alignment of VapC toxins of known structures (PDB codes mentioned in the figure) from sub-cluster2 is shown. Fully conserved residues are shown in red and conservatively substituted residues are shown in yellow columns. Secondary structure elements of the first structure are depicted on top of the alignment and corresponding solvent accessibility at the bottom with dark blue implying solvent accessible regions, cyan showing partially accessible regions and white showing inaccessible regions. High conservation can be appreciated from the alignment. Aligned VapC structures are shown in the inset. (**B**) Alignment of VapB antitoxins of known structures. Same colour scheme as in (**A**) is followed. Alignments were rendered using ESPript [25]. Antitoxins show poor sequence conservation. Aligned VapB structures are shown in the inset.

To understand the structural basis of regulation of cross-talk among *M. tuberculosis* TA systems, we first analyzed the experimentally solved structures in detail. Based on the sequence similarity between VapC toxin sequences in *M. tuberculosis*, we had earlier proposed that they may be grouped into sub-clusters suggesting close relationships among few TA systems [20]. Thus, it is reasonable to hypothesize that the toxins and the antitoxins within a sub-group have the potential to interact with non-cognate partners, provided they are expressed in the cell at the same time. To test this hypothesis, we employed the sub-cluster alignments derived from our earlier study. The second largest sub-cluster (i.e., sub-cluster2) (Figure S1) from that study was considered for analyses because experimentally determined structures are available for at least three TA pairs (VapBC2, PDB code: 3h87; VapBC11, PDB code: 6a7v; and VapBC15, PDB code: 4chg) and a toxin (VapC21, PDB code: 5sv2) [21,22,23,24]. An alignment of the toxins revealed high structural and sequence similarity (Figure 1A) but the alignment between the antitoxins showed low sequence conservation and poor structural alignment (Figure 1B). 

The interfaces of three of these TA systems were compared to probe for similarities in their interacting modes (Table 1). 

**Figure 2:**
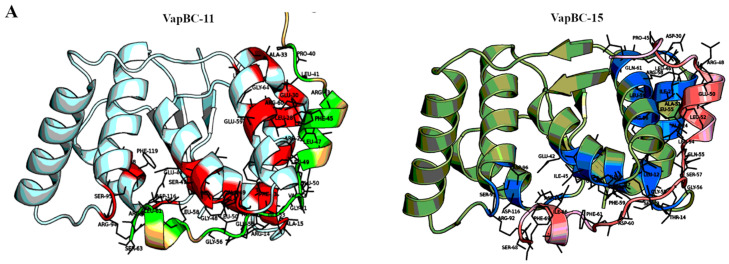

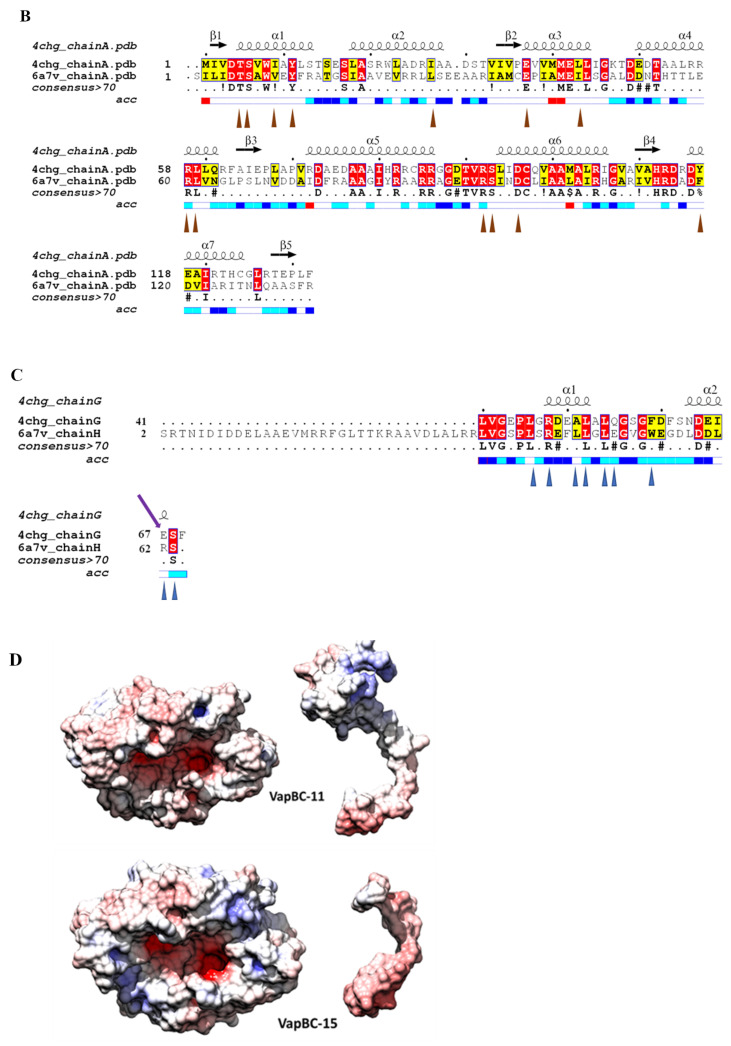
Comparison of VapBC11 and VapBC15 interfaces. (**A**) Cartoon rendering of VapBC11 (6a7v) and VapBC15 (4chg) structures. VapC11 toxin is shown in cyan, VapB11 antitoxin in wheat and residues lining the interfaces in red for VapC11 and light-green for VapB11. VapC15 toxin is shown in dark-green, VapB15 antitoxin in light-pink colour, residues lining VapBC15 interface in blue for VapC15 toxin and in dark-red color for VapB15 antitoxin. Residues at the interface are labelled for clarity. (**B**) Structure-guided pairwise alignment for VapC15 (4chg:A) and VapC11 (6a7v:A). Secondary structural elements of VapC15 are shown on top of the alignment and corresponding solvent accessibility at the bottom. Fully conserved residues are shown in red and conservatively substituted residues are shown in yellow columns. Residues marked with triangles are the topologically equivalent, antitoxin-binding residues in VapC15 and VapC11. (**C**) Structure-guided pairwise alignment for VapB15 (4chg:G) and VapB11 (6a7v:H). Here also, residues marked with triangles are topologically equivalent, toxin-binding residues in VapB15 and VapB11. Interface residue comparisons in Section 2.6 suggest that the residue position marked with a purple arrow (E67) is the likely specificity conferring site in VapB15. Alignments were rendered using ESPript [25]. (**D**) The electrostatic potential surface for VapBC11 is shown on the top and for VapBC15 is shown at the bottom. Dark blue implying solvent accessible regions, cyan showing partially accessible regions, and white showing inaccessible regions. Both toxins and antitoxins show high similarity in their potential surface, suggesting similar surface interactions. The contouring of the surfaces is between ±10 kBT/e.

It was observed that out of the three toxin–antitoxin systems from sub-cluster2, only VapBC11 and VapBC15 showed high similarity between their interfaces (IS-score: 0.52). The structures of VapBC11and VapBC15 are shown in Figure 2A with interface residues marked as sticks in different colour. A pairwise sequence comparison between the toxins VapC11 and VapC15 shows conservation of many antitoxin-binding residues (Figure 2B). Interestingly, a pairwise alignment between antitoxins VapB11 and VapB15 also shows conservation of a majority of the toxin binding residues (Figure 2C). Since our results suggest the possibility of cross-talk between VapBC11 and VapBC15, we compared the electrostatic potential surfaces of both toxins and antitoxins (Figure 2D).

VapC11 and VapC15 showed similar electrostatic surfaces. Likewise, their cognate antitoxins VapB11 and VapB15 also showed similarities in their surfaces but with some differences observed at the C-terminus, as shown by change in charge distribution (discussed in Section 2.6). Therefore, non-cognate TA complexes (VapB11–VapC15 and VapB15–VapC11) were in-silico generated based on VapBC15 and VapBC11 as templates (Figure S2). Side-chains were optimized and structures were energy minimized. The calculated interface similarity score showed high similarity in the interfaces between cognate VapBC15 and modelled VapB11–VapC15 (Is-score: 0.57) and cognate VapBC11 and modelled VapB15–VapC11 (IS-score: 0.54) (Table 2). Interestingly, for the non-cognate pair VapB11–VapC15, the interaction energy (−19.1 Kcal/mol) was found to be comparable to the interaction energy of the cognate VapBC15 TA pair (−21.0 Kcal/mol). This suggests that the modelled non-cognate TA pair was reliable with no clashes between the toxins and antitoxins. However, it is important to note that the interaction energy for the non-cognate pair VapB15–VapC11 was found to be −10.7 Kcal/mol, as compared to −35.5 Kcal/mol in VapBC11. The reasons for this disparity are intriguing given the interface similarity score, as we discuss later. 

### 2.2. Templates Could Be Identified for Toxins at a Higher Confidence than Antitoxins 

Since, very few experimentally determined structures are available for TA systems from *M. tuberculosis*, we attempted computational modelling of those toxins and antitoxins for which no experimental structures are available. Multiple sequence alignments of uncharacterized query sequences with homologues of known functions are useful in annotating their potential functional roles. The confidence of predicting functional residues in such proteins increases, if they can be assigned to the same fold associated with the homologues, at high confidence. Since the sequence identity of the toxins and antitoxins with their respective homologues was not high, templates/folds were recognized for the toxins and antitoxins using different approaches (details in Section 5) (Table S1). We observed that while folds could be predicted for toxins, the confidence of association was low for antitoxins. 

As shown in the pie chart in Figure S3A, for 16% of the cases, a known fold could be assigned with high confidence and sequence identity >30%. For 68% of the cases, a known fold could be assigned with high confidence but at sequence identities <30%. We considered such assignments, despite low sequence identities, since our earlier analysis of toxin sequences showed that catalytic residues and few other residues with structural roles are conserved despite low sequence identities [20]. Nine percent of the cases were assigned to a fold with low confidence and sequence identity <30%, and for ~7% of the cases no folds could be assigned. Next, the homology models were generated for individual toxins based on the identified templates (Table S1). The aim was to enrich the TA sequence data with structural information so that it would further help in identifying and mapping the interfaces of toxin–antitoxin interactions. Nearly 46 VapC toxins and 9 MazF toxins were modelled, with 38 structures reported with appreciable validation scores from ProQ [26] and HARMONY [27].

The results showed that fold assignments for antitoxins was difficult and challenging on account of low sequence conservation (Table S1 and Figure S3B). For most antitoxins the structural assignment was ambiguous, as folds could be recognized only for 37 out of 81 antitoxins with a low confidence. Moreover, since the C-terminal toxin-binding domain of many antitoxins is known to be intrinsically disordered in the absence of toxin, it was observed that those low confidence assignments were further limited to the N-terminal DNA-binding domains. Therefore, it was infeasible to model antitoxins in their free forms and the idea of modelling antitoxins through independent template recognition was not taken forward.

### 2.3. High Confidence Complexes Could Be Generated for Select TA Systems

Since antitoxins could not be modelled in their free form, they were modelled in complex with their cognate toxin partners. We used template-based docking to model the TA complex for six TA pairs (details in Section 5). For these six TA pairs, the templates are toxin–antitoxin systems either from *M. tuberculosis* or other micro-organisms. Details of the queries and their templates along with sequence identity, query coverage, e-values, and shape complementarity are listed in Table S2. The list includes three VapBC (VapBC3, VapBC4, and VapBC21) TA systems and 3 MazEF (MazEF3, MazEF6, and MazEF9) systems. The alignments of the query TA pairs with their templates is shown in Figure S4. The interfaces were extrapolated from the templates. The side-chain optimized, energy minimized structures of the complexes are shown in Figure S5.

### 2.4. Assessment of the Modelled Complexes Reveals Strengths and Limitations of Homology Modelling of TA Systems

Predicted toxin–antitoxin interfaces were assessed for the six modelled TA complexes. It can be observed from Table S2 that while the sequence identity between toxin and template for VapC4, VapC21, MazF3, MazF6, and MazF9 was >25%, it was <20% for VapC3. Among the antitoxin targets and templates, the sequence identities were <20% for VapB3 and MazE3. These sequence identities lie within the twilight zone of 20–30% and hence we considered energy values, structural features, and absence of repulsive interactions to ensure the compatibility of interfaces, in addition to the geometric extrapolation from the templates. To achieve this, the S_c_ score and interaction energy (described in Section 5) were calculated for the modelled structures (Table 3). The S_c_ statistic suggested high shape complementarity for VapBC4, VapBC21, MazEF6, and MazEF9. For MazEF3, the Sc statistic suggested high shape complementarity with one MazF3 chain and low shape complementarity for the other chain. This could have been due to the slight displacement of the antitoxin with respect to the other chain. VapBC3 showed a S_c_ score of 0.59, suggesting that the interface complementarity was not high for the model. S_c_ score for the crystal structures VapBC11, VapBC15, VapBC5, and VapBC2 was calculated as 0.737, 0.739, 0.68, and 0.69, respectively.

While this study was being conducted, crystal structures for toxins VapC21 (PDB code: 5sv2), MazF3 (PDB code: 5uct), MazF6 (PDB code: 5hk0), and MazF9 (PDB code: 5hjz) [29,30] became available in the PDB [24,31]. Therefore, we compared the modelled toxin structures with their crystal structures to assess if these structures could be used to replace the toxins in the modelled TA complexes (Figure S6). RMSD between the modelled and crystal structures are shown in Table S3. Though the global RMSD is small, local structural differences were observed between them, e.g., the difference in conformation of the loop between the β1 and β2 strands (Figure S6). This loop is responsible for the switch between the antitoxin-bound and substrate-bound conformation of MazF toxins [32,33]. An attempt to generate MazEF6 and MazEF9 complexes with the experimental structures resulted in backbone clashes, suggesting that MazF6 and MazF9 were likely to undergo a conformational change in the β - β2 loop region to accommodate the cognate antitoxin, as reported in earlier studies [32,33]. Although MazEF3 and VapBC21 complexes could be generated using the crystal structure of the toxin, multiple rounds of energy minimization were required to remove side-chain clashes indicating the importance of correct side-chain geometry at the interfaces. This showed that modelling of the correct complex is dependent on the conformational state of the template proteins. Hence, it is reasonable to model toxins and antitoxins as pairs, so that the interface regions are correctly identified, which otherwise may not be accessible in the toxin/antitoxin structures alone. To further assess the compatibility of modelled TA pairs, the electrostatic potential surfaces of modelled toxins and antitoxins were compared (Figure S7). For VapBC21 complex, the surface of VapC21 was found to be moderately positively charged, and that of VapB21, which fits into the cavity of VapC21, was found to be moderately negatively charged, and hence is suggestive of the compatibility between VapB21 and VapC21 (Figure S7A). Similarly, the surface of VapC4 and VapB4 was also found to be compatible with each other (Figure S7B). The electrostatic potential surface for MazF3 showed a negatively charged surface patch near the active site and MazE3 showed a positively charged surface patch in its toxin binding region (Figure S7C). In the structure of the complex, these two regions were found to rightly dock with each other. In contrast, the MazF6 and MazF9 structures showed a positively charged patch on the surface, while their cognate antitoxins MazE6 and MazE9 showed a negatively charged patch suggesting compatibility between their toxins and antitoxins surfaces as well (Figure S7D,E). 

### 2.5. Use of High Confidence Modelled Complexes to Explore Cross-Reactivity

Our comparative studies on the interface of modelled TA complexes and their solved structures showed that deriving models for these complexes is non-trivial. Our studies demonstrated that interpretations from the model are best limited to proposing potential interface residues but cannot be extended to reliably predict the binding mode of interaction between toxin and antitoxin. Therefore, we used only the high confidence modelled structures for further analysis. 

Sub-cluster 6 was the other cluster from our grouped toxin alignments that had members with solved crystal structures (Figure S1) [20]. We were unable to derive a reasonably confident model for any of the other cluster members, for the reasons mentioned earlier. Among the members of this sub-cluster, the crystal structure for VapBC5 (PDB code: 3dbo) was available and a high confidence model could be generated for VapBC4 [34]. Before using modelled VapBC4 for the analysis, residues at the interfaces were verified from the existing literature. Mutational analysis has previously shown that residues Asp64, Trp48, Thr66, Leu72, Gln78, Ile55, Leu58, Val59, Leu61, Gly62, Leu 68, Glu71, Glu74, Thr79, Asp81, and Asp82 of VapB4 lie at the VapBC4 interface [35]. In the modelled VapBC4, the majority of these residues lay at the interface improving confidence in further analysis with this model (Table S4). Therefore, we compared the interfaces of VapBC4 and VapBC5 complexes and found high similarity indicated by the IS-score of 0.81 (Table 1 and Figure S8A). A pairwise comparison of VapC4 and VapC5 toxins showed conservation of many antitoxin-binding residues. Similarly, a pairwise comparison of VapB4 and VapB5 antitoxins showed conservation of many toxin-binding residues (Figure S8B,C). The electrostatic potential surfaces of both the Vap toxins and antitoxins were found to be similar (Figure S8D). These results raise the possibility of cross-talk among the two TA systems. Therefore, non-cognate complexes (VapB4–C5 and VapB5–C4) were generated in-silico based on the cognate crystal structure of VapBC4 (Figure S8E). Side-chains for the non-cognate pairs were optimized, and structures were energy minimized. A high similarity was observed between the interface of cognate complex VapBC4 and the modelled VapB5–VapC4, and cognate VapBC5 and the modelled VapB4–VapC5 (Table 2). Intriguingly, for these non-cognate pairs, theoretical binding energy calculations showed comparable energies between VapBC4 and VapB5–C4 (−35 Kcal/mol and −39 Kcal/mol, respectively). In contrast, interaction energy scores for VapB4–C5 was −26 Kcal/mol as compared to −34 Kcal/mol for VapBC5. Theoretically, these results predict a strong binding between the non-cognate pairs. While no experiments have been reported that refute or claim non-cognate interaction between VapC5 and VapB4, Jin et al. demonstrated earlier the inability to rescue growth defect of VapC4 by VapB5 [35]. This motivated us to further probe the discrepancy between computational predictions and experimental results. We also attempted to uncover the reason behind the large difference between interaction energies of the cognate VapBC5 and non-cognate VapC5–VapB4. 

To this end, we analyzed the similarities/differences in interactions at the interface of cognate VapBC5 and VapBC4 complexes to determine if any important hot-spot interactions were lacking in the non-cognate TA models. Hot-spots are residues which when mutated to alanine, change the binding energy of the complex by more than 2 Kcal/mol [36]. Interestingly, we observed that almost all hot-spot interactions were preserved in the non-cognate VapB5–VapC4 and VapB4–VapC5 pairs when compared to cognate VapBC4 and VapBC5 pairs, respectively (Table S4). The only exceptions were the interactions between Arg12 (VapB5) and Glu60 (VapC5) in the VapBC5 complex, and Phe21 and Val14 in the VapBC4 complex (Figure S8C and Table S4). In case of non-cognate VapB5–VapC4 complex, Val14 was substituted by Arg14 and hence formed unfavorable interactions with VapC4. Since the spatial proximity of non-bonded atoms does not have a drastic effect on the overall interaction energy, we obtained comparable energies between the VapBC4 and the VapB5–VapC4 complex. Indeed, Val14 has been shown as critical for VapB4 to rescue the growth defect of VapC4 [35]. This goes on to suggest that interaction energies may not be the only criteria to assess the feasibility of a non-cognate complex and a detailed analysis of the residue properties at the interface is also important. For the non-cognate VapB4–VapC5 complex, it was observed that two negatively charged residues viz., Glu12 of VapB4 and Glu60 of VapC5, came in proximity at the interface. This is the reason behind difference in the binding energies of cognate VapBC5 and non-cognate VapB4–VapC5 and is likely to disrupt the interface. It is known that a single residue mutation can render interactions in a TA complex non-specific [11]. Hence, we believe that Arg12 (VapB5) is a likely specificity conferring residue in the VapBC5 complex and Val14 (VapB4) in the VapBC4 complex. It is to be noted that residue numbers for this case are mentioned according to the residue numbers shown for sequence alignments in Figure S8B,C.

### 2.6. In-Silico Point Mutation of Antitoxin Residues Is Predicted to Relax the Specificity 

Although our analysis predicted that a non-cognate VapB15–VapC11 interaction would be favourable, with a negative interaction energy, it was much weaker than the cognate pair. Therefore, we explored the reasons for these weak interactions. The pairwise alignment between VapB15 and VapB11 showed a small yet significant difference between the topologically equivalent residues; Glu at position 67 in VapB15, and Arg at position 62 in VapB11 (Figure 2C). Residue Glu67 in VapB15 coordinated with the positively charged Mn^2+^ and Mg^2+^ ions in the catalytic site of VapC15. Arg62 in VapC11 (which is solved without a metal ion) bound with the negatively charged aspartate residues. This difference was also reflected in the electrostatic potential surfaces of C-terminals of VapB15 and VapB11 (Figure 2D). We found that because Arg62 in VapB11 could favorably interact with aspartate/glutamate of the active site in VapC15, the interaction energy between VapB11 and VapC15 was close to the cognate VapBC15 pair. However, the negatively charged Glu67 in VapB15 contributed unfavorably to the negatively charged active site of VapC11 toxin, rendering an energetically less favourable non-cognate TA pair in comparison to the cognate VapBC11 TA pair. This led to the identification of a residue position, which was likely the specificity conferring site for VapB15 and was in-silico mutated to Arg67 in VapB15C11 complex (Figure S9A).

In-silico point mutation in FoldX is known to alter only the side chain with no effect on the backbone conformation. Rotamer conformation was optimized and the mutated complex was energy minimized till no further improvement in energy. The rationale behind this mutation was to check if E67R mutation can lower the energy of VapB15–VapC11 complex. Such residues, which contribute strongly to the interaction energies between two proteins, are termed as super-hotspot residues and confer strength and specificity to the protein complex [37]. Indeed, it was observed that E67R mutation contributes favorably and a marked improvement in the interaction energy (−21.7 Kcal/mol) was observed over wild-type VapB15–VapC11 complex (−10.1 Kcal/mol). This difference is well above the error rate of FoldX and can be considered reliable. Hence, it is hypothesized that residue Glu67 was likely a specificity conferring residue in VapB and a single point mutation could relax its specificity. Similarly, for VapB4–VapC5 complex, Glu12 of VapB5 was mutated to Arginine (E12R) (Figure S9B). The mutated complex was energy minimized and the interaction energies were calculated. It was observed that the mutation E12R contributed favorably and further lowered the energy of the (R12)VapB4–VapC5 complex to −30.3 Kcal/mol over the wild-type VapB4–VapC5 complex (−26 Kcal/mol). This lends further support to Arg12 being the specificity conferring residue in VapB5. Indeed, experiments have been performed previously on *H. influenzae* VapBC1/VapBC2, where a single or double mutation in the antitoxin was found to relax the specificity of antitoxin towards its cognate toxin [11]. So, for both non-cognate complexes, we present testable hypothesis, which could be helpful in designing mutations to relax the specificity in TA systems.

### 2.7. Modelled Non-Cognate TA Pairs that Fail to Show Cross-Talk in Experiments Differ in Their Interfaces

**Figure 3:**
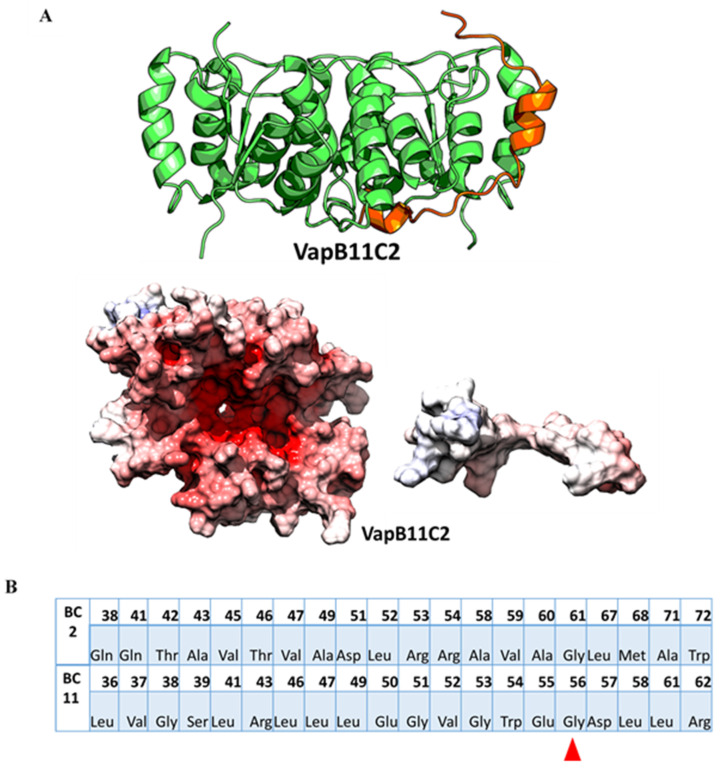
Interface comparison for VapBC complex known to not interact. (**A**) In-silico model for VapB11–VapC2, a non-cognate pair known to not interact is rendered as cartoon. The electrostatic potential surface comparison reveals a strongly negative potential for VapC2 while a less positive or almost neutral surface for VapB11, suggesting incompatibility of the surface. (**B**) shows comparison of the topologically equivalent interface residues in cognate VapB2 and VapB11 antitoxins. Interestingly, the majority of the antitoxin residues at the interfaces show opposite physicochemical properties except for one residue (Gly, red triangle), which is identical among the interface residues contributed by antitoxins.

Ramage et al. demonstrated the inability of select TA systems to cross-react through experiments [10]. However, the growth rescue experiments did not explain the basis for this lack of interaction. We wanted to offer a structural basis of why this interaction was not observed, and therefore performed a computational analysis to be used as control for our study. Out of those few, VapBC2 and VapBC11 belong to the sub-cluster2. Since, the experimental structures are available for VapBC2 and VapBC11, their interfaces were analyzed to explain the basis of the absence of cross-reactivity. As depicted in Figure 1, VapB2 and VapB11 show poor conservation. From Table 1, it can be observed that the interface similarity between VapBC2 and VapBC11 is very low with poor statistical scores. Despite meagre sequence and interface similarity, a brute-force structure for non-cognate VapB11–VapC2 was modelled using VapBC2 structure as template. The electrostatic potential surface and interface comparison of VapC2 and VapB11 showed incompatibility as well (Table 4 and Figure 3). 

A closer inspection of the interface residues contributed by VapB2 and VapB11 revealed conservation of only one residue. Interestingly, the majority of the antitoxin residues at the interfaces showed incompatible physicochemical properties advocating for dissimilarity and incompatible interactions between this non-cognate pair. Similarly, the interface similarity between the non-cognate TA pair VapB2–VapC11 and VapBC11 was also found to be low (Table 4).

### 2.8. MazEF Systems from M. tuberculosis Show Weak Signals for Cross-Reactivity

**Figure 4:**
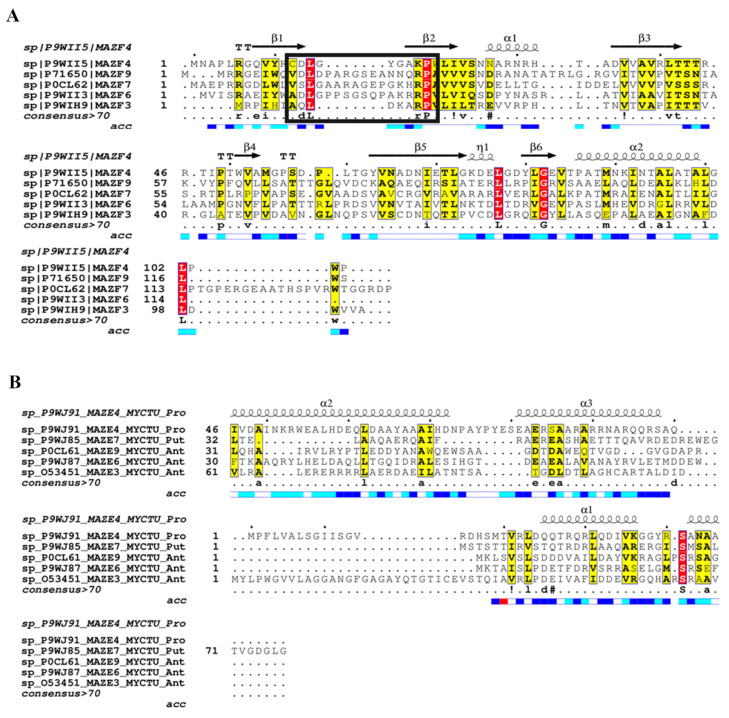
Structure-guided alignments of MazF toxins and MazE antitoxins. (**A**) Structure-guided alignment of MazF toxins show overall high similarity with many residues conserved. Secondary structural elements of MazEF4 are shown on top of the alignment and corresponding solvent accessibility at the bottom with dark blue implying solvent accessible regions, cyan showing partially accessible regions, and white showing inaccessible regions. Fully conserved residues are shown in red and conservatively substituted residues are shown in yellow columns. Sequence of loop region between β1-β2 is marked in black box. (**B**) Structure-guided alignment of MazE antitoxins show less conservation as compared to the toxins. Alignments were rendered using ESPript [25].

There are 10 MazEF TA systems reported in *M. tuberculosis* so far [38]. BLASTP searches revealed that while the majority of the MazF toxins are homologous to each other, their corresponding antitoxins do not identify each other. Hence, unlike VapB antitoxins that could be clustered into small groups [20], MazE antitoxins could not be grouped together. Out of the 10, experimental structures are available for five MazF toxins (MazF3 (PDB code: 5uct), MazF4 (PDB code: 5xe2), MazF6 (PDB code: 5hk0), MazF7 (PDB code: 5wyg), and MazF9 (PDB code: 5hjz)) and two MazEF pairs (MazEF4, PDB code: 5xe3 and MazEF7, PDB code: 6a6x) [16,29,30,31,39]. A structural alignment of MazF toxins revealed high overall similarity among them with few conserved antitoxin-binding residues suggesting similarity in their interfaces (Figure 4A). However, it is known that the length of the loop region between β1 and β2 strands defines the conformational state of MazF protein which is specifically modulated by the cognate antitoxins. Moreover, a structure-guided sequence alignment of MazE antitoxins clearly showed poor residue conservation, suggesting specific interactions with cognate toxins (Figure 4B). 

Our sequence-based analysis therefore does not predict any signals for potential cross-talk among these systems. However, since high confidence complex structures could be modelled for MazEF3, MazEF6, and MazEF9, they were used along with MazEF4 and MazEF7 crystal structures to compare their interfaces (Table 5). It was observed that apart from MazEF3/MazEF4 and MazEF6/MazEF9, all other pairs showed no similarity between their interfaces. For MazEF3/4 and MazEF6/9 also, the similarities (0.45 and 0.38, respectively) were only modest. But since these scores included the contribution from both toxin and antitoxin, and the p-value statistics associated with these scores was good (7.5 × 10^−8^ and 2.4 × 10^−7^, respectively), the cross-talk can only be predicted (though with low confidence) for these two pairs. For the rest, cross-talk seems dubious.

Existence of cross-talk among MazEF TA systems is long debated. Very recently, cross-talk between MazF9 and MazE7 has been reported [16]. Zhu et al. observed non-cognate interactions between MazF6 and MazE9 [13] but this claim was refuted by Ramirez et al. as they could not capture interactions between any MazEF systems [14]. Nevertheless, attempts were made to generate non-cognate MazEF complexes, viz., MazE3–MazF4/MazE4–MazF3 and MazE6–MazF9/MazE9–MazF6. However, all attempts were futile and ended up with very high energy non-cognate complexes primarily due to van der Waal’s clashes. An exception was MazE4–MazF3, which showed interaction energy of −1.95 Kcal/mol between one MazE4–MazF3 chain. However, here too, a high interaction energy was observed for the other chain (2.0 Kcal/mol) likely due to an incompatible toxin/antitoxin conformation with the non-cognate partner. 

## 3. Discussion

Cross-reactivity (or cross-talk) between *M. tuberculosis* toxin–antitoxin systems is anticipated due to presence of paralogous toxin–antitoxin systems and their co-expression in the cell under different stress conditions [40,41]. One would expect these TA systems to be involved in manifold interactions to manage the stress response network of the cell. The delicate balance between toxins and antitoxins regulate the expression of a given TA system [8,42]. It can be expected that if cross-talk exists, the ratio of cognate and non-cognate antitoxins will also be critical for regulating toxin activity. Intriguingly, except for a few dispersed studies, there exists no direct and confident evidence in favor of cross-talk among toxin-antitoxin systems. The present study is an attempt to resolve the existing debate on occurrence of cross-talk among *M. tuberculosis* TA systems from a purely computational point-of-view. The aim of this work is to provide structural insights into the principles behind cognate and non-cognate toxin-antitoxin interactions. A systematic 3D structural analysis has been carried out to explore the feasibility for cross-talk and propose testable hypotheses.

Availability of structures is a prerequisite for such an analysis. However, the experimentally solved structures are available only for a handful of TA complexes or toxins alone in *M. tuberculosis*. Hence, attempts were made to model as many high-confidence structures for TA systems either in complex or free form. Due to low sequence identities (mostly within the twilight zone) between the query toxins or antitoxins with their templates, utmost care was taken to model 46 VapCs and 9 MazF toxins. Antitoxin modelling was especially very difficult due to poor sequence conservation and presence of intrinsic disorder, which limits the applicability of modelling exercise. Further, toxins like MazFs are known to undergo conformational changes upon binding to their cognate antitoxins. These observations prompted us to generate models of toxins and antitoxins in their complexed form rather than as individual toxins or antitoxins. Such attempts may not always yield completely accurate models but provide reasonably accurate representation of the interfaces. Therefore, template-based modelling approach was utilized to model six TA complexes for which the templates and queries showed high similarity for both toxins and antitoxins. We believe that stoichiometry is important for biological function, especially for transcriptional control of TA expression, but the experimental information on this is not available for many TA systems. Since our aim was to analyze the TA interfaces, we kept the stoichiometry for the models to be same as the templates under the assumption that homologous protein–protein complexes are likely to interact in the same manner [43].

Since the TA models were generated using templates with sequence identities between 17% and 34%, they were used to assess the similarity among TA interfaces along with experimental structures with caution. Through a detailed structural analysis, we found that VapBC11–VapBC15 and VapBC4–VapBC5 shared high interface similarity (IS-score: 0.52 and 0.81, respectively). Their electrostatic potential surfaces were found compatible and high similarity was observed between the VapC11/VapC15 (31%), VapB11/VapB15 (52%), VapC4/VapC5 (37%), and VapB4/VapB5 (39%) sequences. In-silico generated non-cognate TA pairs were suggestive of potential cross talk between VapB11–VapC15, VapB4–VapC5, and VapB5–C4 due to comparable interaction energies with VapBC15 (−19.1 Kcal/mol and −21.0 Kcal/mol, respectively), VapBC5 (−26 Kcal/mol and −34 Kcal/mol, respectively), and VapBC4 (−35 Kcal/mol and −39 Kcal/mol, respectively). However, only a low stability structural model (with weak interaction energy) was obtained for VapB15–VapC11. VapB antitoxins are known to block the toxic effect of VapC toxins by binding to their active site and a high compatibility exists between the VapB and VapC electrostatic surfaces in cognate pairs. However, in the case of VapB15/VapC11 non-cognate pairing, this compatibility is compromised by the presence of two negatively charged amino acids at the VapB15/VapC11 interface. So, even though the interaction energy is negative (−10.1 kcal/mol), the two negative charges will repel each other at the active site, and hence we propose that this non-cognate interaction may not be stable enough to neutralize the given toxin. 

Interestingly, we observed that the in-silico point mutation of E67R in the VapB15/VapC11 complex yielded a low energy complex and is predicted to relax the specificity of VapB15. This result provides insights into the reasons for insulation of cross-talk among TA systems. We have earlier reported from the sequence alignments of TAs that while toxins show conservation of many antitoxin binding residues, this does not hold true for antitoxins in each cluster [20]. This was reflected in the interface analysis of VapBC11/VapBC2 systems, which are experimentally shown not to interact with non-cognate pairs. However, in the case of VapBC4/VapBC5, although our theoretical analysis revealed clear and strong signals for cross-reactivity, it has been shown that VapC4 could not be neutralized by VapB5 [35]. Further analysis revealed that even though the theoretical energies were comparable to the cognate complexes, a single residue may be important for maintaining the specificity as shown by marked improvement in the interaction energy of VapB4–C5 non-cognate complex upon mutation of E12R residue in VapB5. Though further experiments can test our hypothesis, this certainly suggests that caution should be exercised while extrapolating the results from theoretical analysis. We summarize that the reasonably comparable and good energy values predicted through modelling efforts need not always imply congenial interactions. These studies must be necessarily accompanied with studies of the interface to recognize scenarios such as drastic changes to charges of residues at the interface, potential short contacts through replacements with sterically incompatible residues, absence of critical hydrogen bond forming residues, or change in van der Waal energies accumulated over many residues at the interface. All these factors can contribute to weaker non-cognate interactions despite satisfactory complementarity or energy scores. It is important to note that in addition to these theoretical estimates, several other factors can govern the failure to observe non-cognate TA interactions. These include factors such as expression of the TA systems. Cognate TAs are known to co-express; however, non-cognate TA need to be expressed at the same time for cross reactivity to occur. Another factor is the stoichiometry, i.e., how many individual units are required for formation of the TA and then for cross-reactivity. The number of toxin and antitoxin molecules present in a studied system can influence the outcome of their interaction, provided they have a compatible interaction. If a non-cognate interaction is predicted to be favorable, factors governing the number of toxin and antitoxin molecules in the system can play a decisive role in determining the experimental outcome of such a prediction. Further, mutation of crucial residues and unstructured regions becoming structured or ordered on physical interaction, can impact the binding strength. Modelling of the TA complex can only estimate the potential interaction energy of the complex and scenarios such as changes to the fold of a protein on binding cannot be suitably accounted. Lastly, the accuracy of computational models relies on sequence identity. Expected accuracies are low when identity of the sequence with its template is low [44].

For the four MazEF complexes (three models and one crystal structures), while the majority of putative non-cognate interfaces showed no interface similarities, only MazE4–MazF3 and MazE9–MazF6 showed weak similarities in their interfaces suggesting potential of cross-talk among these systems. So far, there are no confirmed reports available for cross-talk among MazEF systems in *M. tuberculosis* apart from MazE9–MazF6, which is also debatable. Indeed, absence of cross-talk seems to be favored by nature because even a slight imbalance of toxin–antitoxin systems can be catastrophic for the bacterial cells. While the results here suggest that majority of the non-cognate TA complexes are incompatible, cross-reactivity could be predicted for few. Taken together, this study provides general set of rules and testable hypothesis for cross-talk and further experiments can elucidate if this non-cognate binding among TAs is functional and can neutralize the non-cognate toxin in vitro.

## 4. Conclusions

In the present study, we have employed a computational approach to determine the structural basis for cognate TA interactions and devised rules that are useful to predict cases where non-cognate interactions are feasible for *M. tuberculosis*. We demonstrate, through the case studies of VapBC11–VapBC15 and VapBC4–VapBC5, that in addition to interaction energy values of the predicted non-cognate TA complexes, compatibility of residues at the interaction interface is a key factor in governing the formation of the TA complex (whether cognate/non-cognate as the case may be). We show that in some cases, although interaction interfaces may seem compatible overall and interaction energy scores are favourable, the local environment of residues mediating interaction are critical in predicting whether a non-cognate TA interaction is possible. We elaborate on the structural basis of why non-cognate interactions have failed in earlier experiments that have also attempted to examine this.

## 5. Materials and Methods

### 5.1. Comparative Modelling of Toxins, Antitoxins and TA Complexes

**Figure 5:**
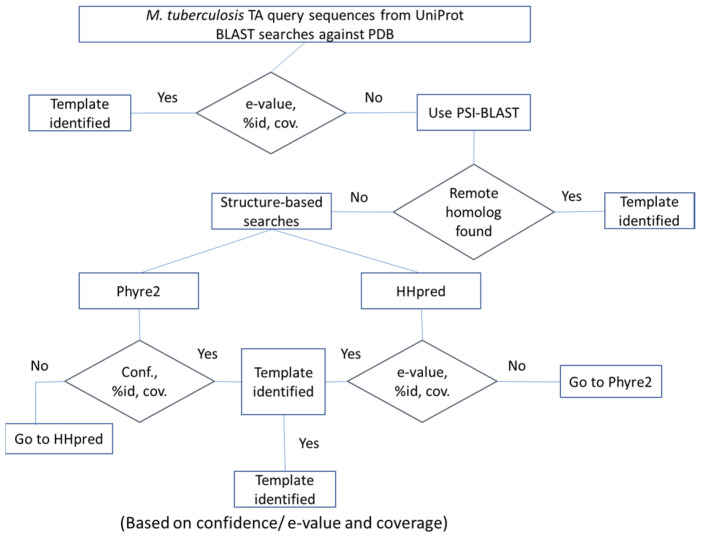
Step-by-step procedure to identify templates for toxins/antitoxins. *M. tuberculosis* TA sequences from UniProt are first searched against PDB using BLAST. If a given hit qualifies the e-value cut-off of 10^−4^ and query coverage >70%, then the template is identified. If no hits are found, PSI-BLAST is used for remote homologue search against PDB. If no template is found, then more comprehensive fold assignment methods such as Phyre2 and HHpred are used, both of which are HMM alignment-based methods.

It is observed that toxins in general share between 16 to 80% sequence identity with their homologues, while antitoxins show poor sequence conservation (20 to 50%) [20]. Consequently, a search for homologues for toxin or antitoxin queries in PDB could not always identify hits that could serve as templates in homology modelling for several of the toxins and antitoxins. Therefore, to identify a suitable template for the toxins or antitoxins, a multi-step procedure was used (Figure 5).

Query toxins and antitoxins from *M. tuberculosis,* for which crystal structure information was not available in the PDB, were identified. Their sequences were obtained from the UniProt database and queried against the protein data bank (PDB) using BLASTP or PSI-BLAST [45]. If a hit was found at an e-value < 10^−4^ and query coverage > 70%, it was selected as a suitable template. If no hits were identified in BLAST searches, rigorous profile HMM-based search strategies, Phyre2 [46] and HHpred [47], were employed to identify a suitable template. A confidence score > 90% from Phyre2 and e-value < 10^−4^ from HHpred were employed as the cut-off criteria. Query coverage >70% was used for both Phyre2 and HHpred. Once the template was identified, Modeller v9.14 was used to model the query protein [48]. Models were generated based on the available co-ordinates deposited in the PDB and only the aligned regions were considered for model building. Since the stoichiometry for many TA systems was not known, it was kept same as the templates while modelling. 100 models were generated per query and the model with lowest DOPE score was selected as final model. Side chains for the final model were optimized using SCWRL4.0 rotamer library [49]. This side-chain optimized model was further subjected to quality check using ProQ [26] and HARMONY [27]. Homology searches for antitoxin sequences did not result in the identification of suitable templates in many instances. Since antitoxins could not be confidently modelled in the unbound form, template-based modelling approach was used to generate the TA complex. In this approach, two proteins, A and B, for which the complex structure was to be modelled, were used as queries in searches for a template. If their respective homologues, A’ and B’, were also known to interact, and their complex structure was readily available, then AB complex was modelled based on A’B’. If this criterion was not met, we did not attempt to build the model for the TA pair AB.

### 5.2. Assessment of Toxin–Antitoxin Interfaces

Depending on the sequence identity, a modelled query complex is anticipated to possess interfacial features like the template. This is the underlying assumption of the approach called template-based modelling (docking) of the protein–protein complexes [50,51,52]. However, this assumption may not always predict correct interfaces. Hence, to obtain high confidence models, the generated complexes were further assessed for interface compatibility using three criteria:

- Surface complementarity was checked using S_c_ statistics, which measures the geometric surface complementarity of protein–protein interfaces [53]. S_c_ depends on the relative shape of the surfaces with respect to each other and on the extent to which the interaction brings individual elements of opposing surfaces into proximity. The score ranges between 0 and 1 and the threshold is generally decided based on the shape complementarity between antigen–antibody interfaces, where the weakest shape complementarity interface is reported with S_c_ values between 0.64 and 0.69.
- Electrostatic surface complementarity was assessed by calculating the electrostatic potential of the proteins. Hydrogens were added to the individual proteins. Charges were assigned to the residues using PDB2PQR [54] plugin and electrostatics calculation was performed using APBS [55] plugin in Chimera 1.13.1. The molecular surface was then color based on electrostatic potential and scaled between ±10 k_B_T and manually inspected.
- Interaction energy between toxin and antitoxin was calculated using AnalyseComplex module from FoldX package [28]. FoldX force-field is empirical in nature with terms for de-solvation energies, coulombic interactions, van der Waal’s forces, hydrogen bonding, entropic changes, and others. All the structures were energy minimized with GROMACS v5.1 using CHARMM27 force-field and steepest descent method for either 50,000 steps or till convergence. A dodecahedron box with TIP3P water molecules was defined around the protein and the system was neutralized by adding counter ions prior to minimization. The structures were further repaired for any distorted geometry using RepairPDB module from FoldX prior to energy calculations. The complex structures were minimized iteratively till no further improvement in the energy values.

### 5.3. Using Structures to Explore Cross-Reactivity between Non-Cognate Toxins and Antitoxins

Toxins cluster into distinct sub-clusters based on their sequence identity [20]. To predict if a non-cognate toxin–antitoxin pair can interact and form a stable complex, we selected the clusters of paralogous toxins with the highest number of known crystal structures for further analysis was chosen. From each of the sub-clusters of 2 and 6, structures of the cognate TA pairs (Figure S1) in each cluster were analyzed and their interfacial regions were compared using iAlign [56]. TA pairs that showed high interface similarity were further probed for cross-reactivity. A pairwise alignment between the toxin and antitoxin sequences of such complexes within each sub-cluster was individually analyzed to identify conserved interactions between cognate TA pairs. In-silico non-cognate complexes were generated with cognate structures as template using Modeller v9.14 [48]. 100 models were generated and side-chains were optimized using SCWRL4.0 [49] rotamer library for the model with least DOPE score. All the in-silico complexes were energy minimized with GROMACS v5.14 as mentioned in the earlier section [57]. Further, interaction energy was calculated, and the shape complementarity of the non-cognate pair was analyzed. Electrostatic potential at the interfaces of non-cognate toxins and antitoxins were compared with cognate TA pairs. As a control, available structures for TA pairs that are experimentally known not to interact were analyzed in a similar fashion to understand the reasons behind insulation of cross-talk between those pairs.

### 5.4. Prediction of Hotspot Residues at the Interface and In-Silico Mutations Using FoldX

After analyzing non-cognate pairs of VapC11–B15 and VapBC4–BC5, residues were identified which could behave as the specificity conferring residue in VapB15 and VapB5. These results were further supported by hotspot predictions using the AlaScan module from the FoldX package [28]. ΔΔG cut-off of 2 Kcal/mol was used. These residues were in-silico mutated using the PositionScan module from the FoldX package. The mutated complex structure was energy minimized and the interaction energies were calculated using AnalyseComplex module of FoldX.

## Figures and Tables

**Table 1:** Interface similarity between different cognate VapBC structures.

Complex Structures to be Compared (Vap)	IS-Score	z-Score	% Seq. Identity	No. of Aligned Contacts	RMSD (Ǻ)	*p*-Value
BC2–BC11	0.25	5	10	24	3.11	3.2 × 10^−3^
BC2–BC15	0.24	5	5	23	3.27	3 × 10^−3^
BC11–BC15	0.52	23	39	48	1.98	4.2 × 10^−11^
BC4–BC5	0.81	45	40	70	0.89	2.5 × 10^−19^

Similarity between the interfaces of VapBC complexes was calculated using iAlign, an interface alignment method for the structural alignment of protein–protein interfaces. IS-score is the interface similarity score, which not only measures geometric distance, but also the conservation of interfacial contact patterns. The higher the IS-score is, the higher is the interfacial similarity. % sequence identity between the interface residues of the two complexes being compared is also provided. The calculations take into consideration, the residue identities from both toxin and antitoxin chains. RMSD gives the deviation between the interface structure. *p*-value assigns the statistical significance to the score (cut-off: 10^−5^).

**Table 2:** Interface similarity between non-cognate VapBC pairs.

Complex Structure	IS-Score	z-Score	% Seq. Identity	No. of Aligned Contacts	RMSD (Å)	*p*-Value	Shape Complementarity for Non-Cognate Pair (S_c_)
BC15–B11C15	0.57	24	70	40	1.62	3.8 × 10^−^^11^	0.67
BC11–B15C11	0.54	23	76	38	1.46	8.3 × 10^−^^11^	0.60
BC4–B5C4	0.81	49	75	79	0.25	2.2 × 10^−^^21^	0.67
BC5–B4C5	0.83	49	83	80	0.23	2.0 × 10^−^^21^	0.65

The higher the IS-score, more is the interfacial similarity. % sequence identity between the interface residues is also provided. The calculations take into consideration the residue identities from both toxin and antitoxin chains. RMSD gives the deviation between the interface structure. *p*-value assigns the statistical significance to the score (cut-off: 10^−5^). The S_c_ statistics measures the geometric surface complementarity of protein-protein interfaces.

**Table 3:** S_c_ statistics and interaction energy values for modelled complexes.

TA System	S_c_ Statistics	Interaction Energy (Kcal/mol)
VapBC3	0.59	-
VapBC4	0.64	−35.04
VapBC21	0.64	−15.83
MazEF3	0.68 & 0.445	−17.2
MazEF6	0.67 & 0.67	−22.2
MazEF9	0.62 & 0.65	−16.2

S_c_ statistics measures the geometric surface complementarity of protein–protein interfaces. The two values for MazEF complexes show the compatibility of each MazF chain with the MazE antitoxin. The interaction energy between toxin and antitoxin was calculated using AnalyseComplex module from FoldX package [28]. A lower interaction energy score of the complex is suggestive of a more stable complex.

**Table 4:** Interface similarity between non-cognate VapBC pairs known not to interact.

Complex Structure	IS-Score	z-Score	% Seq. Identity	RMSD (Å)	*p*-Value	Shape Complementarity (Sc)
BC2–B11C2	0.3	9.5	No residue from antitoxin	2.9	7 × 10^−5^	0.60
BC11–B2C11	0.25	5.7	No residue from antitoxin	3.18	3.3 × 10^−3^	0.59

IS-score is the interface similarity score, which not only measures geometric distance, but also the conservation of interfacial contact patterns. The higher the IS-score, more is the interfacial similarity. *p*-value assigns the statistical significance to the score (cut-off: 10^−5^).

**Table 5:** Interface similarity between cognate MazEF pairs.

Complex Structure to be Compared (Maz)	IS-Score	z-Score	% Seq. Identity	No. of Aligned Contacts	RMSD (Å)	*p*-Value
EF4-EF6	0.16	1.5	35	7	2.38	1.9 × 10^−1^
EF4-EF3	0.45	16.3	30	18	2.39	7.5 × 10^−8^
EF4-EF7	0.12	−0.4	10	3	1.78	0.8 × 10^−1^
EF4-EF9	0.16	0.6	0	8	2.75	3.9 × 10^−1^
EF3-EF6	0.15	0.84	25	4	2.32	3.4 × 10^−1^
EF3-EF7	0.13	0.34	11	4	3.52	5.0 × 10^−1^
EF6-EF7	0.20	3.75	4	12	3.00	2.3 × 10^−2^
EF6-EF9	0.38	15.3	7	20	1.56	2.4 × 10^−7^
EF3-EF9	0.14	0.12	0	8	3.81	5.8 × 10^−1^
EF7-EF9	0.18	2.67	20	13	3.13	6.6 × 10^−2^

Interface similarity was calculated using iAlign. IS-score is the interface similarity score. Higher the IS-score, more is the interfacial similarity. % sequence identity between the interface residues is also provided. The calculations take into consideration the residue identities from both toxin and antitoxin chains. RMSD gives the deviation between the interface structure. P-value assigns the statistical significance to the score (cut-off: 10^−5^).

## Supplementary Materials

The following are available online at https://www.mdpi.com/2072-6651/12/8/481/s1, Figure S1: sub-cluster alignments, Figure S2: in-silico modelled non-cognate VapBC complexes, Figure S3: fold assignment/template prediction for toxins and antitoxins, Figure S4: alignment of query toxins and antitoxins with their templates, Figure S5: homology models for toxin–antitoxin complexes, Figure S6: superposition of modelled and crystal structures of different MazF toxins, Figure S7: electrostatic potential surfaces for toxin–antitoxin models, Figure S8: comparison of VapBC5 and VapBC4 interfaces, Figure S9: in-silico point mutations in VapBs enhance binding, Table S1: template information for TA systems in *M. tuberculosis*, Table S2: details of templates and query TA complexes, Table S3: RMSD between modelled and crystal structures, and Table S4: hotspot interactions between cognate and non-cognate VapBC4–BC5 pairs.

## References

[1] Hayes F., Van Melderen L. (2011). Toxins-Antitoxins: Diversity, Evolution and Function. Crit. Rev. Biochem. Mol. Biol..

[2] Kedzierska B., Hayes F. (2016). Emerging Roles of Toxin-Antitoxin Modules in Bacterial Pathogenesis. Molecules.

[3] Wen Y., Behiels E., Devreese B. (2014). Toxin-Antitoxin Systems: Their Role in Persistence, Biofilm Formation, and Pathogenicity. Pathog. Dis..

[4] Lobato-Márquez D., Díaz-Orejas R., García-del Portillo F. (2016). Toxin-Antitoxins and Bacterial Virulence. FEMS Microbiol. Rev..

[5] Coray D.S., Wheeler N.E., Heinemann J.A., Gardner P.P. (2017). Why so Narrow: Distribution of Anti-Sense Regulated, Type I Toxin-Antitoxin Systems Compared with Type II and Type III Systems. RNA Biol..

[6] Leplae R., Geeraerts D., Hallez R., Guglielmini J., Drze P., Van Melderen L. (2011). Diversity of Bacterial Type II Toxin-Antitoxin Systems: A Comprehensive Search and Functional Analysis of Novel Families. Nucleic Acids Res..

[7] Van Melderen L., De Bast M.S. (2009). Bacterial Toxin-Antitoxin Systems: More than Selfish Entities?. PLoS Genet..

[8] Goeders N., Van Melderen L. (2013). Toxin-Antitoxin Systems as Multilevel Interaction Systems. Toxins.

[9] Park J.H., Yoshizumi S., Yamaguchi Y., Wu K.P., Inouye M. (2013). ACA-Specific RNA Sequence Recognition Is Acquired via the Loop 2 Region of MazF MRNA Interferase. Proteins Struct. Funct. Bioinform..

[10] Ramage H.R., Connolly L.E., Cox J.S. (2009). Comprehensive Functional Analysis of *Mycobacterium tuberculosis* Toxin-Antitoxin Systems: Implications for Pathogenesis, Stress Responses, and Evolution. PLoS Genet..

[11] Walling L.R., Butler J.S. (2016). Structural Determinants for Antitoxin Identity and Insulation of Cross Talk between Homologous Toxin-Antitoxin Systems. J. Bacteriol..

[12] Yang M., Gao C., Wang Y., Zhang H., He Z.G. (2010). Characterization of the Interaction and Cross-Regulation of Three *Mycobacterium tuberculosis* RelBE Modules. PLoS ONE.

[13] Zhu L., Sharp J.D., Kobayashi H., Woychik N.A., Inouye M. (2010). Noncognate *Mycobacterium tuberculosis* Toxin-Antitoxins Can Physically and Functionally Interact. J. Biol. Chem..

[14] Ramirez M.V., Dawson C.C., Crew R., England K., Slayden R.A. (2013). MazF6 Toxin of *Mycobacterium tuberculosis* Demonstrates Antitoxin Specificity and Is Coupled to Regulation of Cell Growth by a Soj-like Protein. BMC Microbiol..

[15] Riffaud C., Pinel-Marie M.L., Pascreau G., Felden B. (2019). Functionality and Cross-Regulation of the Four SprG/SprF Type I Toxin-Antitoxin Systems in *Staphylococcus aureus*. Nucleic Acids Res..

[16] Chen R., Tu J., Tan Y., Cai X., Yang C., Deng X., Su B., Ma S., Liu X., Ma P. (2019). Structural and Biochemical Characterization of the Cognate and Heterologous Interactions of the MazEF-Mt9 TA System. ACS Infect. Dis..

[17] Wei Y., Li Y., Yang F., Wu Q., Liu D., Li X., Hua H., Liu X., Wang Y., Zheng K. (2017). Physical and Functional Interplay between MazF 1Bif and Its Noncognate Antitoxins from *Bifidobacterium longum*. Appl. Environ. Microbiol..

[18] Smith A.B., López-Villarejo J., Diago-Navarro E., Mitchenall L.A., Barendregt A., Heck A.J., Lemonnier M., Maxwell A., Díaz-Orejas R. (2012). A Common Origin for the Bacterial Toxin-Antitoxin Systems ParD and Ccd, Suggested by Analyses of Toxin/Target and Toxin/Antitoxin Interactions. PLoS ONE.

[19] Berman H.M., Westbrook J., Feng Z., Gilliland G., Bhat T.N., Weissig H., Shindyalov I.N., Bourne P.E. (2005). The Protein Data Bank. Struct. Bioinform..

[20] Tandon H., Sharma A., Wadhwa S., Varadarajan R., Singh R., Srinivasan N., Sandhya S. (2019). Bioinformatic and Mutational Studies of Related Toxin–Antitoxin Pairs in *Mycobacterium tuberculosis* Predict and Identify Key Functional Residues. J. Biol. Chem..

[21] Min A.B., Miallau L., Sawaya M.R., Habel J., Cascio D., Eisenberg D. (2012). The Crystal Structure of the Rv0301-Rv0300 VapBC-3 Toxin-Antitoxin Complex from *M. tuberculosis* Reveals a Mg2+ Ion in the Active Site and a Putative RNA-Binding Site. Protein Sci..

[22] Deep A., Tiwari P., Agarwal S., Kaundal S., Kidwai S., Singh R., Thakur K.G. (2018). Structural, Functional and Biological Insights into the Role of *Mycobacterium tuberculosis* VapBC11 Toxin–Antitoxin System: Targeting a TRNase to Tackle Mycobacterial Adaptation. Nucleic Acids Res..

[23] Das U., Pogenberg V., Subhramanyam U.K.T., Wilmanns M., Gourinath S., Srinivasan A. (2014). Crystal Structure of the VapBc-15 Complex from *Mycobacterium tuberculosis* Reveals a Two-Metal Ion Dependent Pin-Domain Ribonuclease and a Variable Mode of Toxin-Antitoxin Assembly. J. Struct. Biol..

[24] Jardim P., da Silva Santos I.C., Barbosa J.A.R.G., de Freitas S.M., Valadares N.F. (2016). Crystal Structure of VapC21 from *Mycobacterium tuberculosis* at 1.31 Å Resolution. Biochem. Biophys. Res. Commun..

[25] Robert X., Gouet P. (2014). Deciphering Key Features in Protein Structures with the New ENDscript Server. Nucleic Acids Res..

[26] Wallner B., Elofsson A. (2003). Can Correct Protein Models Be Identified?. Protein Sci..

[27] Pugalenthi G., Shameer K., Srinivasan N., Sowdhamini R. (2006). HARMONY: A Server for the Assessment of Protein Structures. Nucleic Acids Res..

[28] Schymkowitz J., Borg J., Stricher F., Nys R., Rousseau F., Serrano L. (2005). The FoldX Web Server: An Online Force Field. Nucleic Acids Res..

[29] Yen T.J., Brennan R.G. (2017). Crystal Structure of *M. tuberculosis* MazF-Mt3 (Rv1991c) in Complex with RNA. Ph.D. Thesis.

[30] Yen T.J., Brennan R.G. (2017). Structure of *M. tuberculosis* MazF-Mt1 (Rv2801c) in Complex with RNA. ACS Infect. Dis..

[31] Hoffer E.D., Miles S.J., Dunham C.M. (2017). The Structure and Function of *Mycobacterium tuberculosis* MazF-Mt6 Toxin Provide Insights into Conserved Features of MazF Endonucleases. J. Biol. Chem..

[32] Zorzini V., Mernik A., Lah J., Sterckx Y.G.J., De Jonge N., Garcia-Pino A., De Greve H., Verse W., Loris R. (2016). Substrate Recognition and Activity Regulation of the *Escherichia Coli* MRNA Endonuclease MazF. J. Biol. Chem..

[33] Kamada K., Hanaoka F., Burley S.K. (2003). Crystal Structure of the MazE/MazF Complex: Molecular Bases of Antidote-Toxin Recognition. Mol. Cell.

[34] Miallau L., Faller M., Chiang J., Arbing M., Guo F., Cascio D., Eisenberg D. (2009). Structure and Proposed Activity of a Member of the VapBC Family of Toxin-Antitoxin Systems. J. Biol. Chem..

[35] Jin G., Pavelka M.S., Butler J.S. (2015). Structure-Function Analysis of VapB4 Antitoxin Identifies Critical Features of a Minimal VapC4 Toxin-Binding Module. J. Bacteriol..

[36] Thorn K.S., Bogan A.A. (2001). ASEdb: A Database of Alanine Mutations and Their Effects on the Free Energy of Binding in Protein Interactions. Bioinformatics.

[37] Vishwanath S., Sukhwal A., Sowdhamini R., Srinivasan N. (2017). Specificity and Stability of Transient Protein–Protein Interactions. Curr. Opin. Struct. Biol..

[38] Sala A., Bordes P., Genevaux P. (2014). Multiple Toxin-Antitoxin Systems in *Mycobacterium tuberculosis*. Toxins.

[39] Chen R., Tu J., Liu Z., Meng F., Ma P., Ding Z., Yang C., Chen L., Deng X., Xie W. (2017). Structure of the MazF-Mt9 Toxin, a TRNA-Specific Endonuclease from *Mycobacterium tuberculosis*. Biochem. Biophys. Res. Commun..

[40] Agarwal S., Tiwari P., Deep A., Kidwai S., Gupta S., Thakur K.G., Singh R. (2018). System-Wide Analysis Unravels the Differential Regulation and in Vivo Essentiality of Virulence-Associated Proteins B and C Toxin-Antitoxin Systems of *Mycobacterium tuberculosis*. J. Infect. Dis..

[41] Gupta A., Venkataraman B., Vasudevan M., Gopinath Bankar K. (2017). Co-Expression Network Analysis of Toxin-Antitoxin Loci in *Mycobacterium tuberculosis* Reveals Key Modulators of Cellular Stress. Sci. Rep..

[42] Frampton R., Aggio R.B.M., Villas-Bôas S.G., Arcus V.L., Cook G.M. (2012). Toxin-Antitoxin Systems of *Mycobacterium smegmatis* Are Essential for Cell Survival. J. Biol. Chem..

[43] Aloy P., Ceulemans H., Stark A., Russell R.B. (2003). The Relationship between Sequence and Interaction Divergence in Proteins. J. Mol. Biol..

[44] Yazhini A., Srinivasan N. (2020). How Good Are Comparative Models in the Understanding of Protein Dynamics?. Proteins Struct. Funct. Bioinform..

[45] Altschul S.F., Madden T.L., Schäffer A.A., Zhang J., Zhang Z., Miller W., Lipman D.J. (1997). Gapped BLAST and PSI-BLAST: A New Generation of Protein Database Search Programs. Nucleic Acids Res..

[46] Kelley L.A., Mezulis S., Yates C.M., Wass M.N., Sternberg M.J.E. (2015). The Phyre2 Web Portal for Protein Modeling, Prediction and Analysis. Nat. Protoc..

[47] Hildebrand A., Remmert M., Biegert A., Söding J. (2009). Fast and Accurate Automatic Structure Prediction with HHpred. Proteins Struct. Funct. Bioinform..

[48] Webb B., Sali A. (2016). Comparative Protein Structure Modeling Using MODELLER. Curr. Protoc. Bioinforma..

[49] Krivov G.G., Shapovalov M.V., Dunbrack R.L. (2009). Improved Prediction of Protein Side-Chain Conformations with SCWRL4. Proteins Struct. Funct. Bioinform..

[50] Baspinar A., Cukuroglu E., Nussinov R., Keskin O., Gursoy A. (2014). PRISM: A Web Server and Repository for Prediction of Protein-Protein Interactions and Modeling Their 3D Complexes. Nucleic Acids Res..

[51] Kundrotas P.J., Zhu Z., Janin J., Vakser I.A. (2012). Templates Are Available to Model Nearly All Complexes of Structurally Characterized Proteins. Proc. Natl. Acad. Sci. USA.

[52] Rajagopala S.V., Sikorski P., Kumar A., Mosca R., Vlasblom J., Arnold R., Franca-Koh J., Pakala S.B., Phanse S., Ceol A. (2014). The Binary Protein-Protein Interaction Landscape of *Escherichia coli*. Nat. Biotechnol..

[53] Lawrence M., Colman P. (1993). Shape Complementarity at Protein–Protein Interfaces. J. Mol. Biol..

[54] Dolinsky T.J., Nielsen J.E., McCammon J.A., Baker N.A. (2004). PDB2PQR: An Automated Pipeline for the Setup of Poisson-Boltzmann Electrostatics Calculations. Nucleic Acids Res..

[55] Baker N.A., Sept D., Joseph S., Holst M.J., McCammon J.A. (2001). Electrostatics of Nanosystems: Application to Microtubules and the Ribosome. Proc. Natl. Acad. Sci. USA.

[56] Gao M., Skolnick J., Rost B. (2011). IAlign: A Method for the Structural Comparison of Protein-Protein Interfaces. Bioinformatics.

[57] Christen M., Hünenberger P.H., Bakowies D., Baron R., Bürgi R., Geerke D.P., Heinz T.N., Kastenholz M.A., Kräutler V., Oostenbrink C. (2005). The GROMOS Software for Biomolecular Simulation: GROMOS05. J. Comput. Chem..

