# Supplementary material for: Molecular and Structural Basis of Cross-Reactivity in M. tuberculosis Toxin–Antitoxin Systems
Source: Toxins (Basel). 2020 Jul 29;12(8):481. doi: 10.3390/toxins12080481 (PMC7472061; doi:10.3390/toxins12080481)
Supplement: Supplementary file 1 [file toxins-12-00481-s001.zip › toxins-819408 sp 2/toxins-819408-SI_proof.docx]

Supplementary Materials: Molecular and Structural Basis of Cross-Reactivity in *M. tuberculosis* Toxin–Antitoxin Systems

Himani Tandon, Akhila Melarkode Vattekatte, Narayanaswamy Srinivasan and Sankaran Sandhya


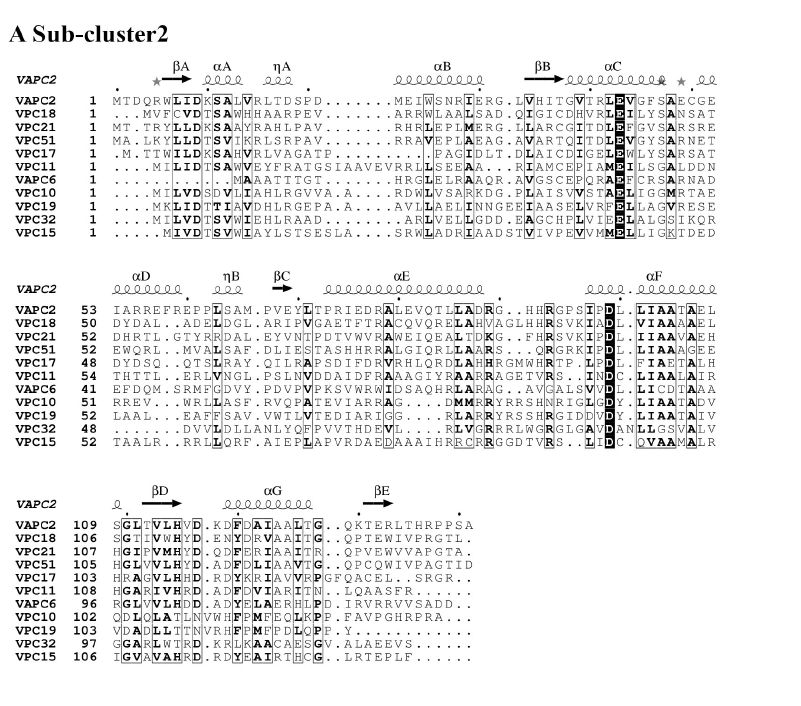


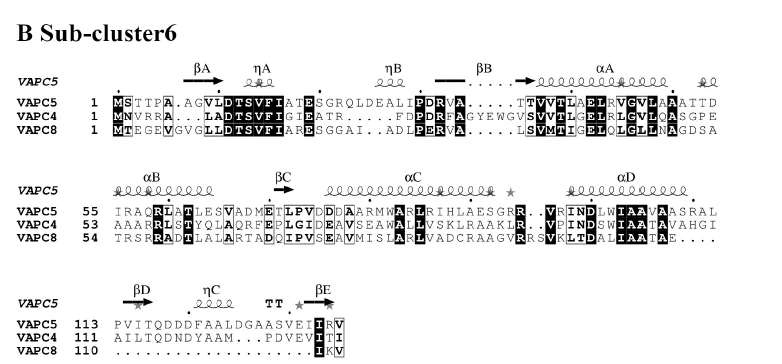


**Figure S1.** Sub-cluster alignments. Multiple sequence alignments for VapBC TA sequences in (**a**) sub-cluster2 (**b**) sub-cluster6 that were analyzed for conserved residue positions in [1]. Members of these sub-clusters have been used to perform the structural analyses in this study. The dark colored columns show conserved residues. Secondary structure elements of the representative structure are shown on top of each alignment.


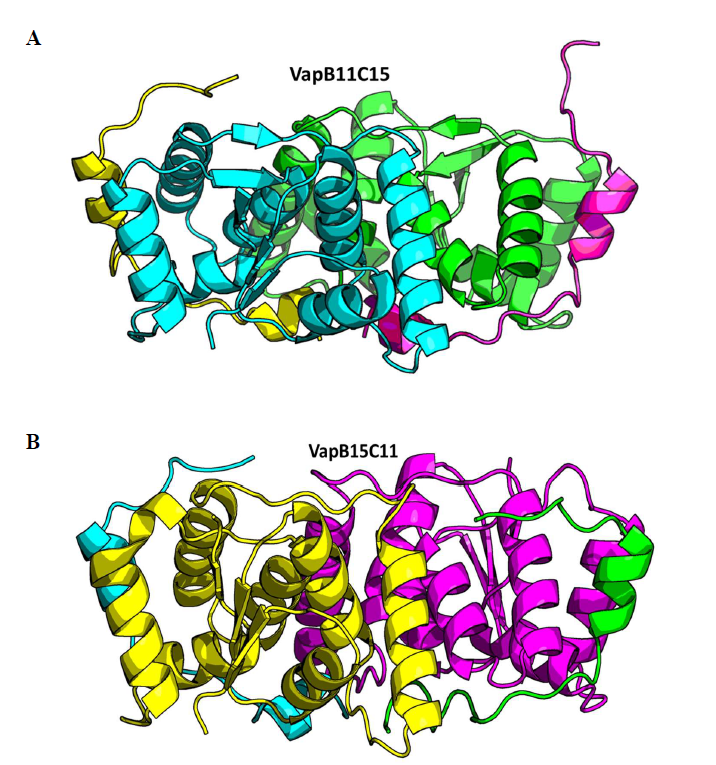


**Figure S2*.*** In-silico modelled non-cognate VapBC complexes. Non-cognate VapB11- VapC15 complex in (**a**) and non-cognate VapB15-VapC11 in (**b**) were modelled using cognate pairs as templates. The two toxin chains are colored as cyan and green complex and two antitoxin chains are colored as pink and yellow in VapB11-VapC15. Color scheme is reversed for VapB15-VapC11 complex. These complexes are sidechain optimized and energy minimized upon modelling.


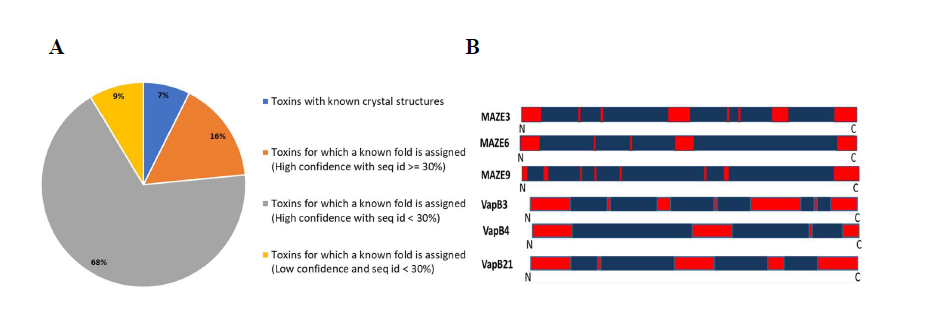


**Figure S3.** Fold assignment/template prediction for toxins and antitoxins. (**a**) Pie-chart showing number of cases for which a known template could be assigned for the toxin sequences, either with high or low confidence. **(b**) Cartoon representation of disordered regions (marked in red) in antitoxins due to which fold assignment for antitoxins is dubious.


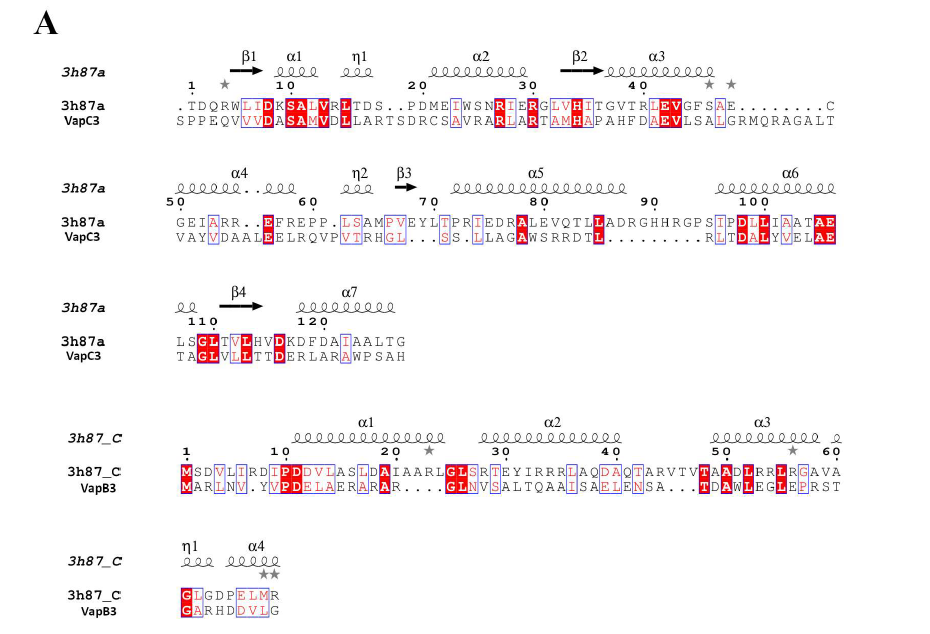


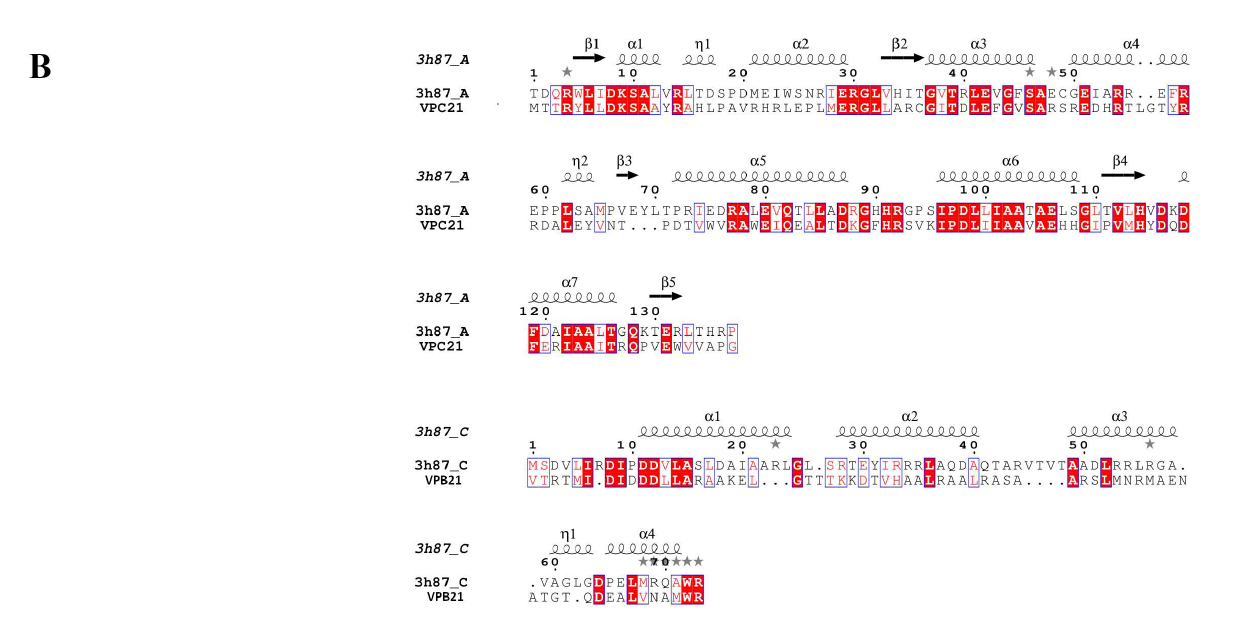


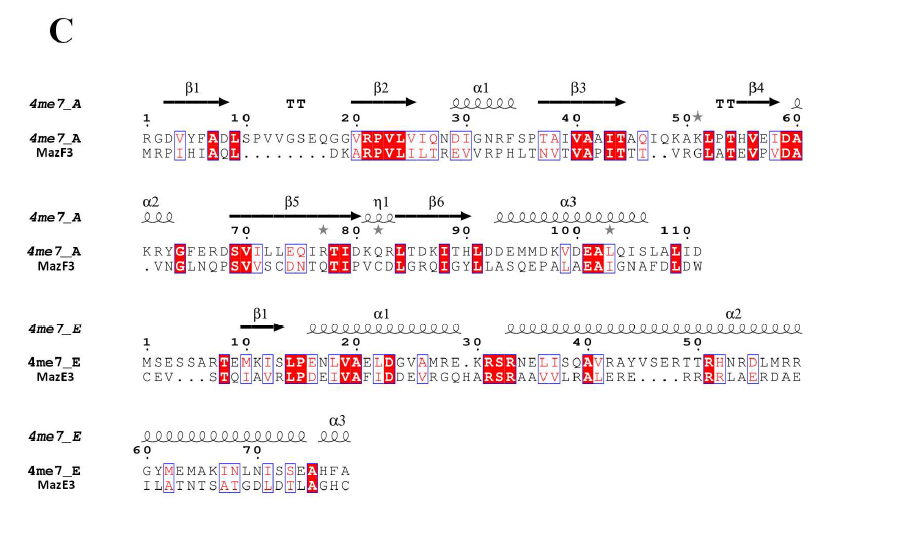


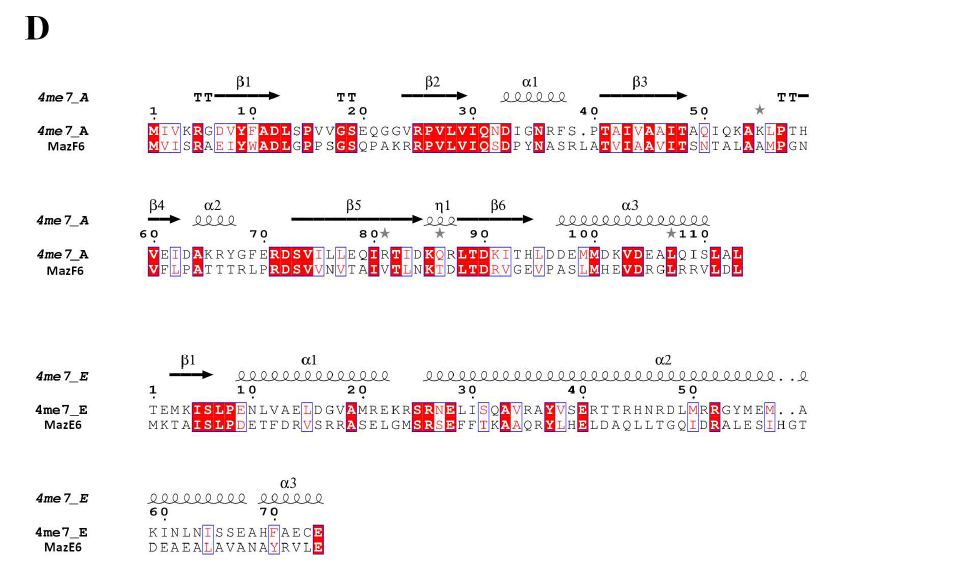


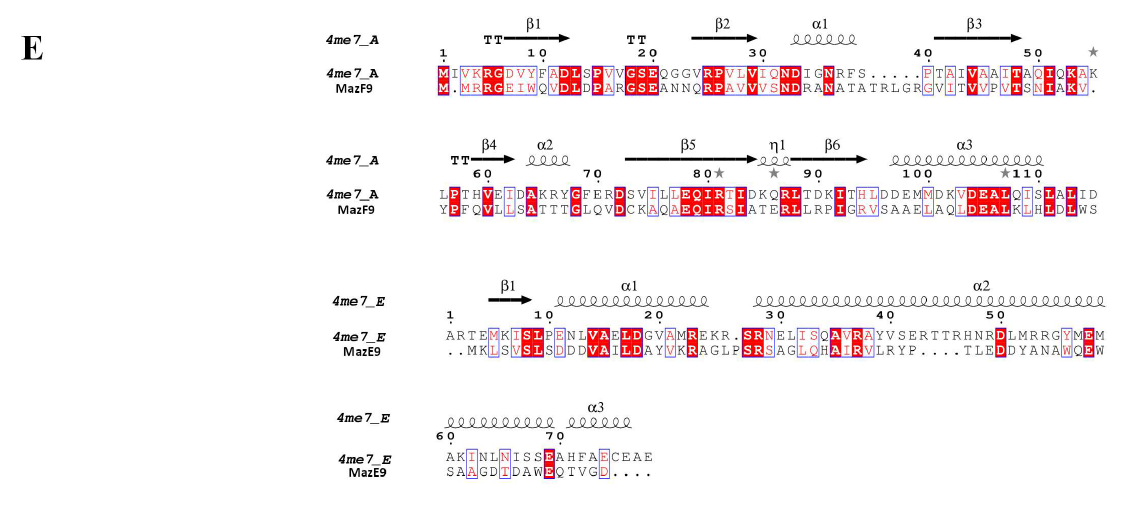


**Figure S4.** Alignment of query toxin and antitoxin sequences with their templates. Structure-guided sequence alignment of target (**A**) VapC3 and VapB3 (**B**) VapC21 and VapB21 (**C**) MazF3 and MazE3 (**D**) MazF6 and MazE6 and (**E**) MazF9 and MazE9 with their respective templates (PDB codes shown in the figure) [2,3]. Identical residues are colored red and conservatively substituted residues are marked in a box. Secondary structure of the template is depicted at the top of each alignment. The alignments were generated using Promals3D [4] and rendered using ESpript [5].


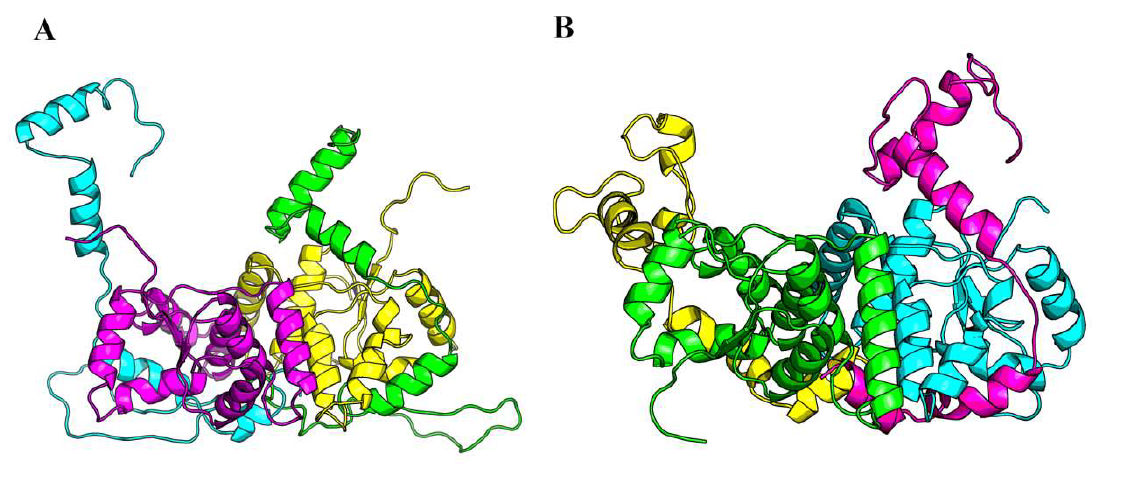


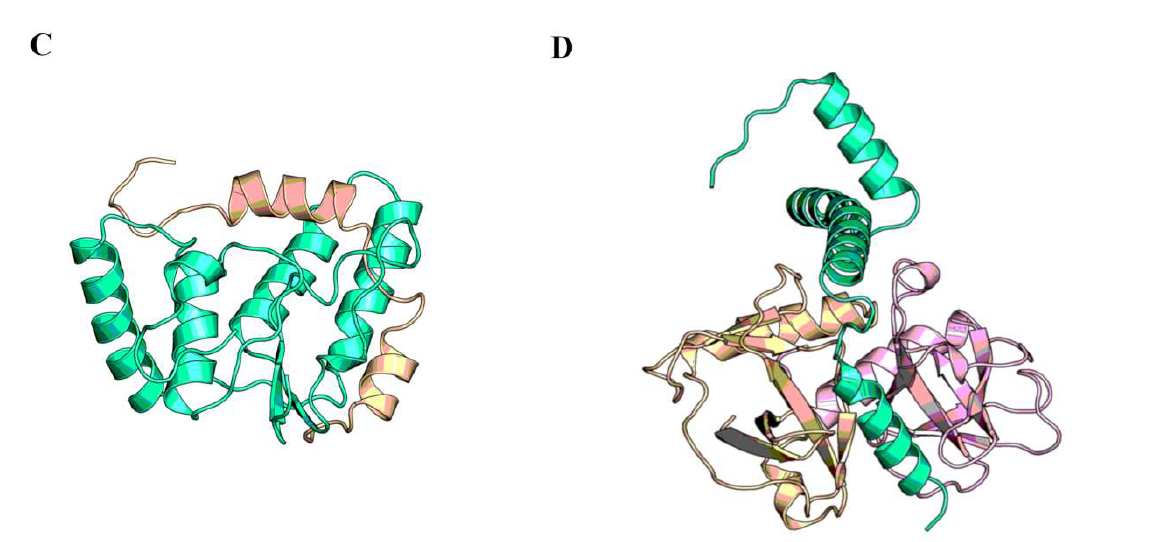


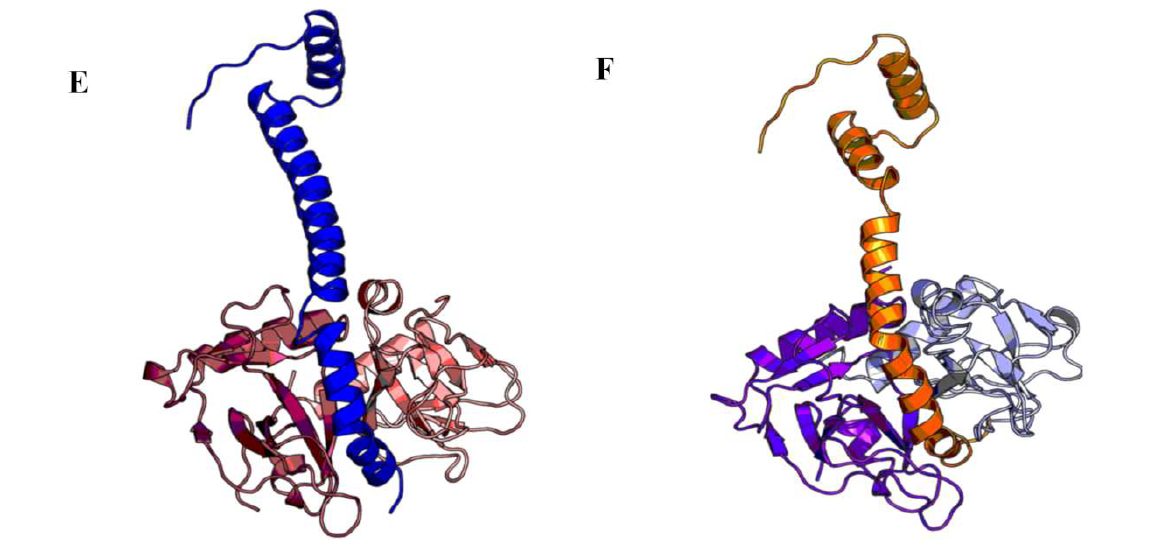


**Figure S5.** Homology models for toxin-antitoxin complexes. Cartoon renderings for homology models of (**a**) VapBC3 (VapC3: pink and yellow, VapB3: green and cyan) (**b**) VapBC21 (VapC21: green and cyan, VapB21: pink and yellow) (**c**) VapBC4 (VapC4: teal green, VapB4: wheat) (**d**) MazEF3 (MazF3: pink and wheat, MazE3: teal green) (**e**) MazEF6 (MazF6: Dark salmon and pink, MazF6: blue) and (**f**) MazEF9 (MazF9: Purple and light blue, MazE9: orange). These complexes have been modelled using crystal structure templates of other TA complexes as templates. Stoichiometry for the modelled structures was derived from the templates.


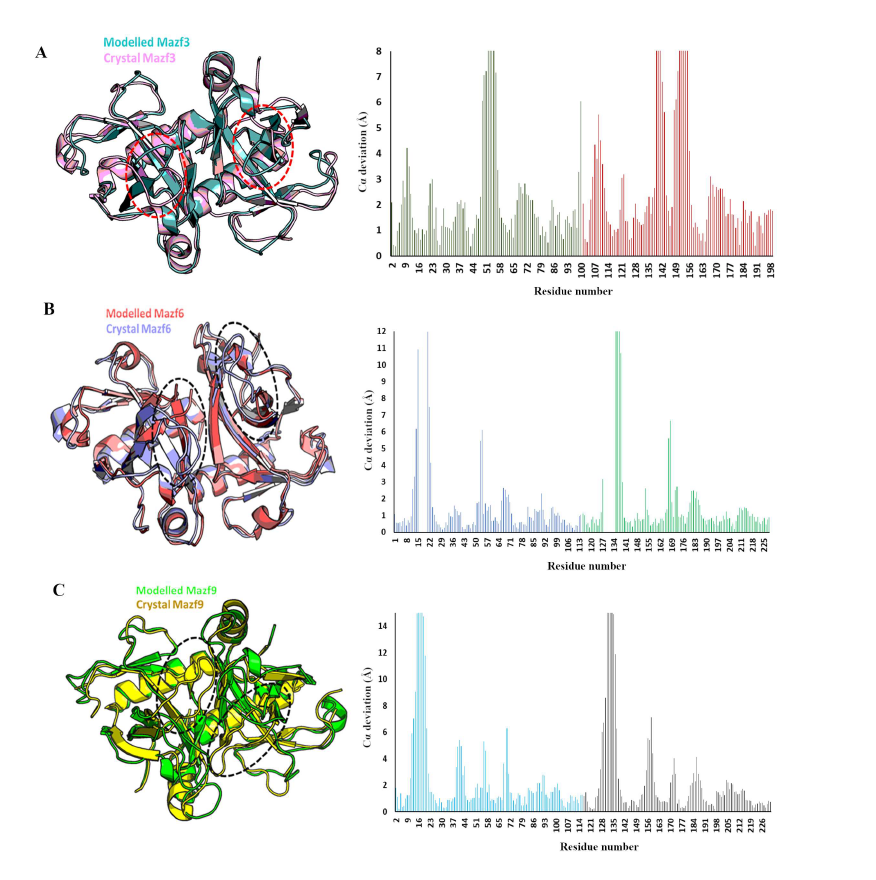


**Figure S6.** Superposition of modelled and crystal structures of different MazF toxins. (**a**) Superposition of modelled and crystal structure of MazF3 (PDB code: 5uct) show differences due to short loop between strand β1-β2 (encircled in red). (**b**) Superposition of modelled MazF6 on MazF6 crystal structure (PDB code: 5hk0) showing differences in the loop between β1-β2 are marked with a black circle. (**c**) Superposition of modelled MazF9 (PDB code: 5hjz) on MazF9 crystal structure shows long loop between β1-β2. The crystal structure of MazF9 shows closed loop conformation as opposed to model, which is modelled as open loop to bind the antitoxin. Residue-wise Cα deviation for each of these comparisons is provided as bar plot. Empty points on the plot indicate missing regions in the crystal structures and correspond to the β1-β2 loop in case of MazF6. It is to be noted that residues flanking the missing regions tend to show very high Cα-deviation. For MazF3 and MazF9, longer bars show high structural deviation and correspond to conformational change in the β1-β2 loop.


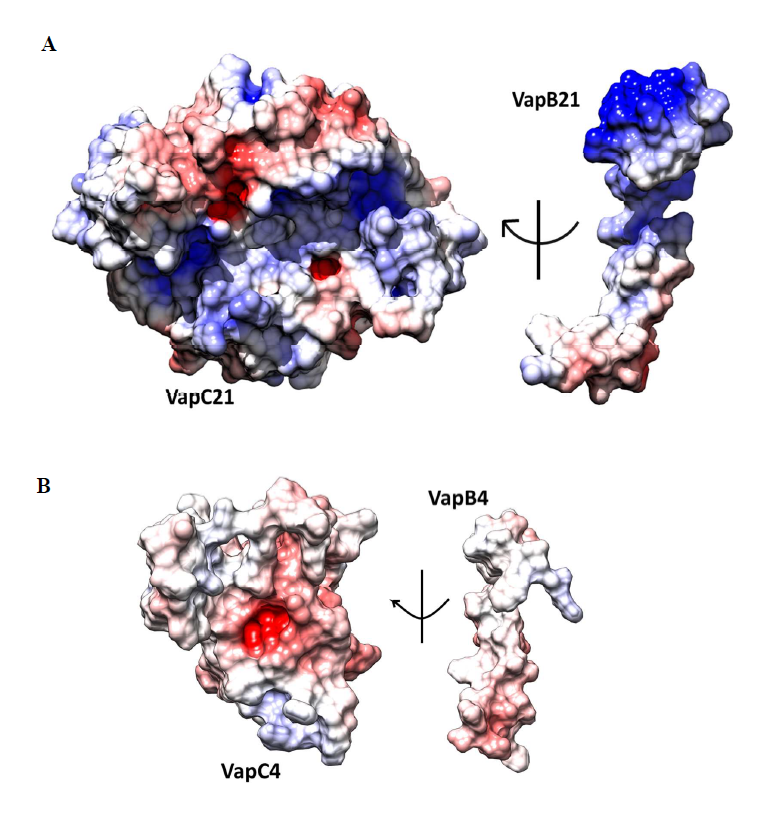


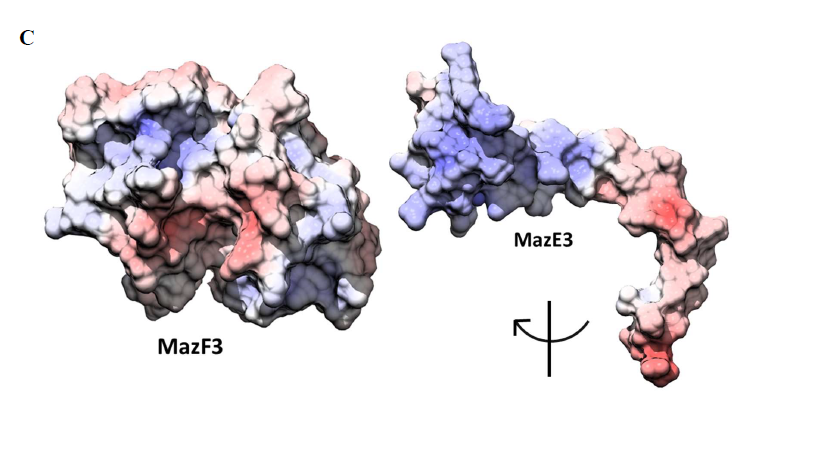


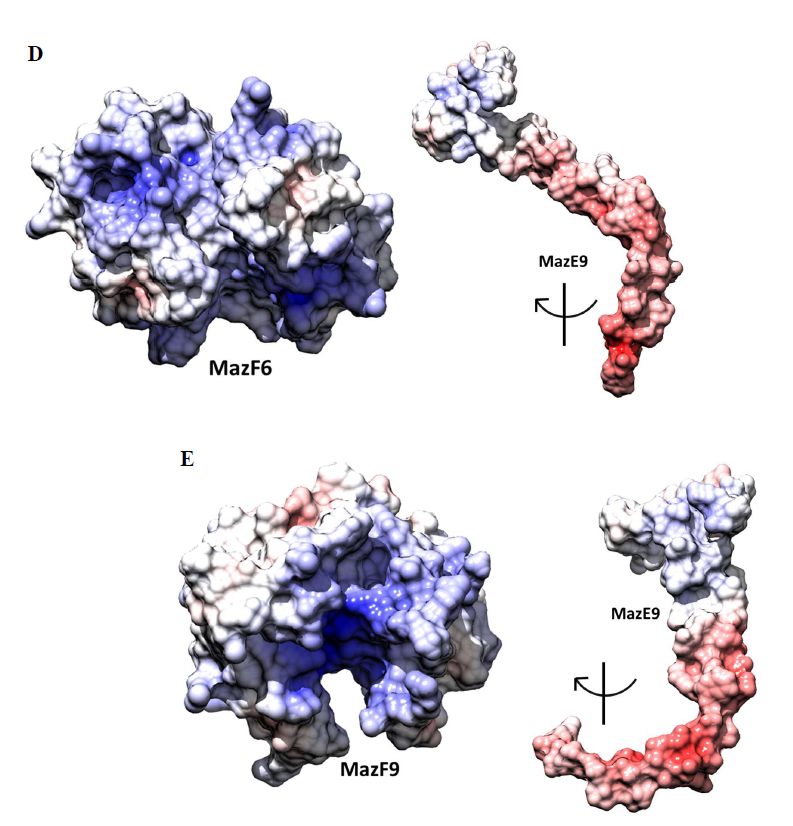


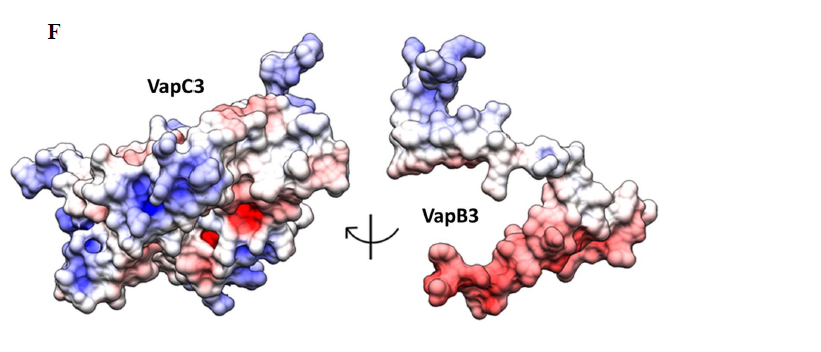


**Figure S7.** Electrostatic potential surfaces for toxin-antitoxin models. Electrostatic potential calculated using APBS and rendered using Chimera for (**a**) VapBC21, (**b**) VapBC4, (**c**) MazEF3, (**d**) MazEF6 (**e**) MazEF9 and (**f**) VapBC3. Contouring for VapBC21 surface is between ±5 kBT/e and for others is between ±10 kBT/e. The direction in which antitoxins were rotated to fit into corresponding toxins is shown with the arrow. All the surfaces show electrostatic compatibility to correctly fit with each other.


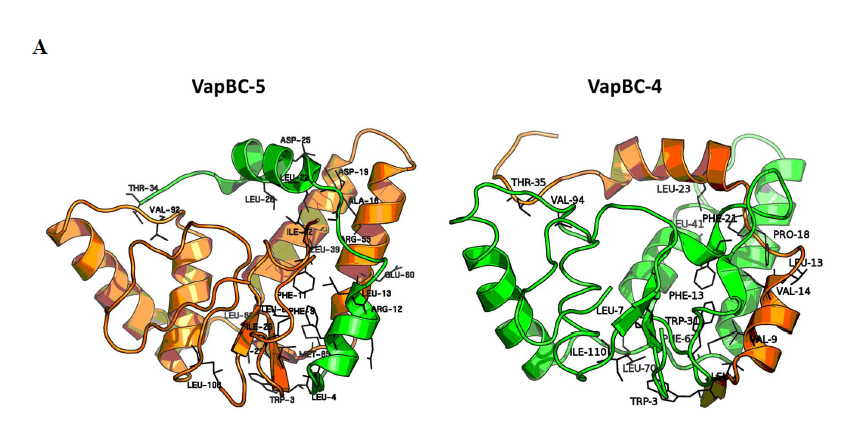


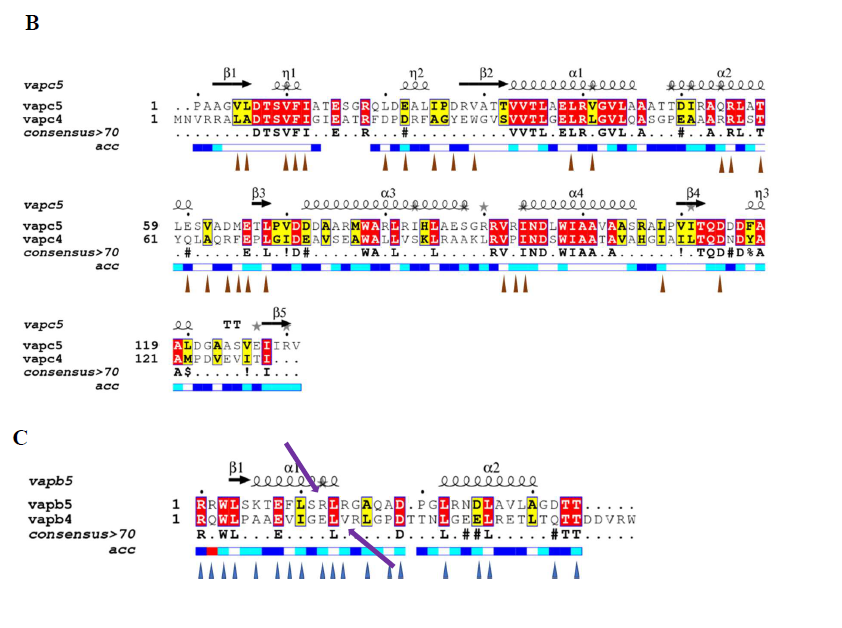


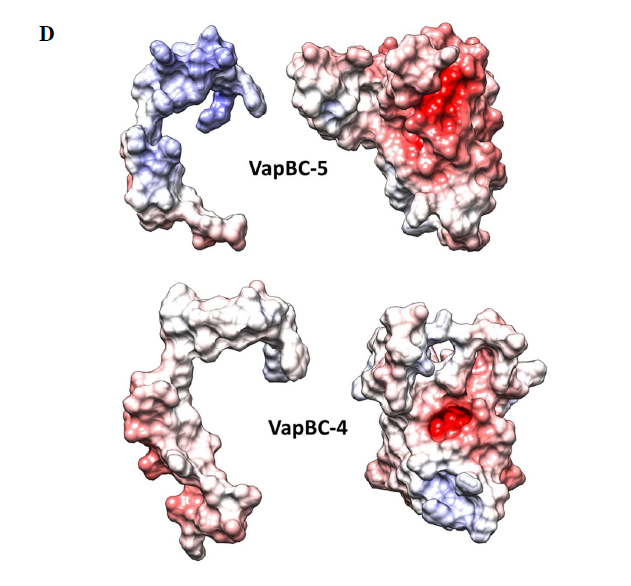


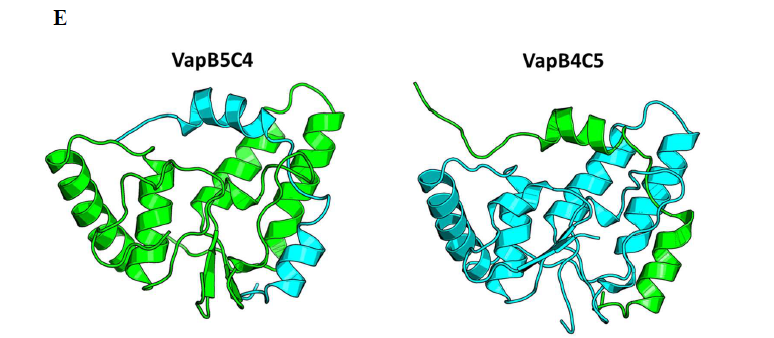


**Figure S8.** Comparison of VapBC5 and VapBC4 interfaces. (**a**) Cartoon renderings of VapBC5 (3dbo) and modelled VapBC4 structures. VapC5 toxin is shown in orange and VapB5 antitoxin in purple color. Residues lining the interfaces are shown as sticks. Similarly, VapC4 toxin is shown in green and VapB4 antitoxin in wheat color. Residues lining VapBC4 interface are shown as sticks. (**b**) Structure-guided pairwise alignment for VapC5 and VapC4 is depicted. Structural elements of VapC5 are shown on the top of the alignment and solvent accessibility at the bottom. Color scheme is as in Figure 2. Residues marked with triangles are the topologically equivalent, antitoxin-binding residues in VapC5 and VapC4. (**c**) Structure-guided pairwise alignment for VapB5 and VapB4 is depicted. Here also, the residues marked with triangles are topologically equivalent, toxin-binding residues in VapB5 and VapB4. The residue position marked with purple arrows are predicted to be the specificity conferring site in VapB5 and VapB4. (**d**) Electrostatic potential surface representations for VapBC5 and VapBC4. (**e**) Non-cognate VapB5-VapC4 and VapB4-VapC5 were modelled using VapBC5 (PDB code: 3dbo) as templates. The toxin chain is colored green and antitoxin chain is colored cyan VapB5-VapC4. Color scheme is reversed for VapB4-VapC5 complex. These complexes are side-chain optimized and energy minimized upon modelling.


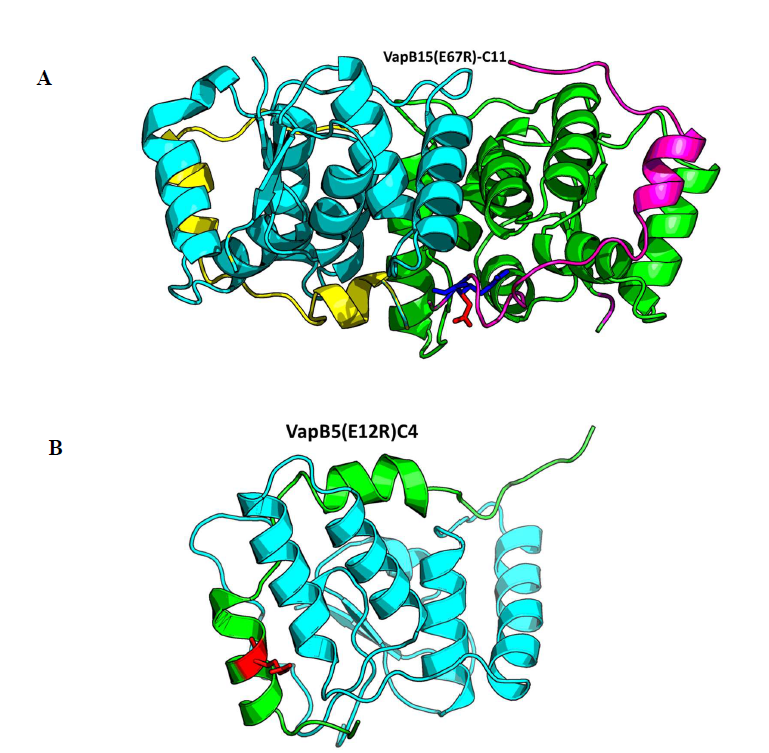


**Figure S9.** In-silico point mutations in VapBs enhance binding. (**a**) A point mutation of VapB15 Glu67 to Arg67 is depicted in the modelled structure. Glu67 (shown in red) points outward in the VapB15C11 structure due to repulsion of negative charges at the interface. Mutation to Arg67 (shown in blue), likely places the side chain into the active site thereby, contributing favorably to the binding. (**b**) Mutation of Glu12 to Arg12 is depicted for VapB5C4 structure. Arg12 after mutation is shown in red.

**Table S2.** Details of templates and query TA complexes. The templates were identified using three approaches namely, BLASTP, Phyre2 and HHpred. A condition that query toxin and antitoxin sequences should identify the same protein complex as their template was imposed. Sc-score provides a measure for the shape complementarity between the toxin and antitoxin. The two numbers for MazEF templates show that the antitoxin MazE forms interface with two MazF chains.

| **TA System** |  |  | **Template** | **Sequence ID**  **(%)** | **Aligned Region** | **e-Value** | **Sc-score for Templates** |
| --- | --- | --- | --- | --- | --- | --- | --- |
| VapBC3 | Toxin | VapC3 | 3h87 | 17 | 7–134 | 4.3 × 10^−15^ | 0.73 |
|  | Antitoxin | VapB3 | 3h87 | 18 | 17–77 | 6 × 10^−4^ |  |
| VapBC4 | Toxin | VapC5 | 3dbo | 36 | 1–147 | 2.9 × 10^−19^ | 0.72 |
|  | Antitoxin | VapC5 | 3dbo | 30 | 1–85 | 6.5 × 10^−11^ |  |
| VapBC21 | Toxin | VapC21 | 3h87 | 39 | 1–136 | 4 × 10^−22^ | 0.73 |
|  | Antitoxin | VapB21 | 3h87 | 23 | 14–80 | 2.9 × 10^−6^ |  |
| MaZEF3 | Toxin | MazF3 | 4mdx/4me7 | 26 | 1–100 | 5 × 10^−30^ | 0.68 & 0.63 |
|  | Antitoxin | MazE3 | 4me7 | 17 | 27–98 | 4 × 10^−11^ |  |
| MaZEF6 | Toxin | MazF6 | 4mdx/4me7 | 38 | 1–114 | 9 × 10^−36^ | 0.68 & 0.63 |
|  | Antitoxin | MazE6 | 4me7 | 18 | 1–76 | 4.6 × 10^−15^ |  |
| MaZEF9 | Toxin | MazF9 | 4mdx/4me7 | 34 | 1–118 | 1.6 × 10^−33^ | 0.68 & 0.63 |
|  | Antitoxin | MazE9 | 4me7 | 21 | 1–69 | 4.6 × 10^−15^ |  |

**Table S3.** RMSD between modelled and crystal structures.

| **TA System** | **RMSD (Ǻ) between Modelled and Crystal Structure** | **TA System** | **RMSD (Ǻ) between Modelled and Crystal Structure** |
| --- | --- | --- | --- |
| VapC21 | 1.79 | MazF6 | 1.47 |
| MazF3 | 2.08 | MazF9 | 1.78 |

**Table S4.** Hotspot interactions between cognate and non-cognate VapBC4-BC5 pairs. The residue number corresponds to the sequence alignment shown in Figure S8. The left residue number corresponds to the toxin and the right to the antitoxin. Some residues can interact with more than one residue at the interface and these are grouped in parenthesis. The important hotspot interactions, which were not found conserved, are marked in bold.

| **TA System** | **Hotspot Interactions at the Interface (Toxin-Antitoxin)** |
| --- | --- |
| VapBC5 | L6-F9; F11-(F9,L13,A18); I12-(A18,L22,L26); I25-F9; V29-(L4,F9); L39-L22; R55-(D19,D25); **E60-R12**; M65-(L4,F9); L68-W3; V92-T34; L108-W3 |
| VapB4C5 | L6-V9; F11-L13; I12-(P18,L27); I25-(A6,I10); V29-V9; L39-(P18,23L); R55-D19, M65-(L4,V9); L68-W3, V92-T35; L108;W3 |
| VapBC4 | L7-W3; F13-P18, L13; **F21-(V14**,L13); W31-(L4,V9); L41-P18,L23; F67-(L4,V9); L70-W3; V94-T35; I110-W3 |
| VapB5C4 | L7-W3; F13-L13; W31-(L4,F9); L41-(P20,L22); F67-(L4, F9); L70-W3; V94-T34, I110-W3 |

References:

1. Tandon, H.; Sharma, A.; Wadhwa, S.; Varadarajan, R.; Singh, R.; Srinivasan, N.; Sandhya, S. Bioinformatic and Mutational Studies of Related Toxin–Antitoxin Pairs in Mycobacterium Tuberculosis Predict and Identify Key Functional Residues. *J. Biol. Chem.*, **2019**, *294*, 9048–9063. https://doi.org/10.1074/jbc.RA118.006814.
2. Min, A.B.; Miallau, L.; Sawaya, M.R.; Habel, J.; Cascio, D.; Eisenberg, D. The Crystal Structure of the Rv0301-Rv0300 VapBC-3 Toxin–Antitoxin Complex from M. Tuberculosis Reveals a Mg2+ Ion in the Active Site and a Putative RNA-Binding Site. Protein Sci., **2012**, *21*, 1754–1767. https://doi.org/10.1002/pro.2161.
3. Simanshu, D.K.; Yamaguchi, Y.; Park, J.H.; Inouye, M.; Patel, D.J. Structural Basis of MRNA Recognition and Cleavage by Toxin MazF and Its Regulation by Antitoxin MazE in Bacillus Subtilis. *Mol. Cell*, **2013**, *52*, 447–458. https://doi.org/10.1016/j.molcel.2013.09.006.
4. Pei, J.; Kim, B.; Grishin, N.V. PROMALS3D: A Tool for Multiple Protein Sequence and Structure Alignments. **2008**, *36*, 2295–2300. https://doi.org/10.1093/nar/gkn072.
5. Robert, X.; Gouet, P. Deciphering Key Features in Protein Structures with the New ENDscript Server. Nucleic Acids Res., **2014**, *42* (W1). https://doi.org/10.1093/nar/gku316.
